# Signatures in the Protein Content of Human and Murine Blood Serum Exosomes, in the Context of Major Depressive Disorder, Are Associated with Cytokine Activity

**DOI:** 10.3390/cells15121042

**Published:** 2026-06-06

**Authors:** Jorge Manuel Vásquez-Pérez, Mónica Flores-Ramos, María del Pilar Ramos-Godínez, Gerardo Bernabé Ramírez-Rodríguez

**Affiliations:** 1 Laboratorio de Neurogénesis, Dirección y Subdirección de Investigación Biomédica en Salud Mental, Instituto Nacional de Psiquiatría Ramón de la Fuente Muñiz, Mexico City 14370, Mexico; mvasquez@ciencias.unam.mx; 2 Programa de Doctorado en Ciencias Biomédicas, Facultad de Medicina, Universidad Nacional Autónoma de México, Mexico City 04510, Mexico; 3 Laboratorio de Epidemiología Clínica, Dirección y Subdirección de Investigación Biomédica en Salud Mental, Instituto Nacional de Psiquiatría Ramón de la Fuente Muñiz, Mexico City 14370, Mexico; monica.flores@inprf.gob.mx; 4 Departamento de Microscopía Electrónica, Instituto Nacional de Cancerología, Mexico City 14080, Mexico; mramosg@incan.edu.mx

**Keywords:** depression, major depressive disorder, stress, CUMS model, extracellular vesicles, exosomes

## Abstract

**Highlights:**

**What are the main findings?**

- Human and murine exosomes share protein content in major depressive disorder and chronic mild stress.
- The content of proteins in human and murine exosomes is related to the cytokine activity.

**What are the implications of the main findings?**

- Human exosomes as a source of potential biomarkers in major depression.
- Cytokine signaling proteins may be involved in the effects of exosomes in major depression.

**Abstract:**

Major depressive disorder (MDD) is among the most common and disabling psychiatric disorders. MDD is multifactorial, influencing the central nervous, endocrine, and immune systems. Also, MDD impacts neurochemical and inflammatory pathways via shared signaling mechanisms, including metabolites, soluble factors, and extracellular vesicles (EVs, including exosomes). Here, we hypothesized that EVs from MDD patients or mice exposed to chronic unpredictable mild stress (CUMS) contain specific inflammatory signatures that may help explain the pathophysiology of that mental disorder. We included four groups: healthy female controls (n = 8), women with MDD (n = 12), healthy Balb/C female mice (n = 10), and Balb/C mice under CUMS (n = 10). We isolated and characterized exosome-enriched EVs from human and murine serum and analyzed their protein content using antibody arrays. We identified three protein sets with significant differences (*p* < 0.05): 36 human exosome proteins decreased in the MDD group; 18 murine exosome proteins decreased in the CUMS group; and 12 proteins showed differential expression between human and murine exosomes, mostly trending downward in the CUMS and MDD groups. We performed bioinformatic analysis to determine protein–protein interactions and gene ontology functions. We identified signaling pathways associated with MDD and chronic stress: chemokine, cytokine–cytokine receptor, JAK-STAT, pathways in cancer, Rap1, Ras, TNF signaling, and cytokine interactions. These findings highlight the importance of human and murine exosomes as critical sources for understanding depression’s molecular mechanisms.

## 1. Introduction

Major depressive disorder (MDD) is one of the most prevalent and disabling psychiatric disorders worldwide [1,2,3]. MDD is characterized by persistent negative mood and thoughts accompanied by cognitive impairment, excessive fatigue, and alterations in appetite, libido, and sleep, as described in the *Diagnostic and Statistical Manual of Mental Disorders* (DSM-5) [2,4]. Approximately 5–10% of the global adult population experiences MDD [3,5]. Despite its high prevalence, depression is a complex and multifactorial disorder involving diverse biological mechanisms, including neurotransmitter dysregulation, structural brain alterations, changes in the hypothalamic–pituitary–adrenal (HPA) axis, neuro-inflammation, and molecular signaling abnormalities [6,7,8,9]. Another important aspect in the study of major depressive disorder is the marked sex-related difference in its prevalence and clinical presentation [10,11]. Epidemiological studies consistently report that women are affected by MDD approximately twice as frequently as men, particularly during the reproductive years [2,3,5]. This increased susceptibility has been associated with hormonal fluctuations in estrogen and progesterone, which can influence neurotransmission, neuro-inflammatory responses, and HPA axis regulation [12,13]. These biological factors may contribute to differences in stress responses and vulnerability to depressive disorders in women [14]. For this reason, focusing on women may provide clinically relevant information associated with depression.

Consequently, the pathophysiology of MDD remains incompletely understood and requires integrative approaches that consider sex-related biological, cellular, molecular, and psychological mechanisms contributing to its development. In this context, animal models of stress have become essential tools for investigating the neurobiological mechanisms underlying depressive disorders [15,16,17]. Among these models, the chronic unpredictable mild stress (CUMS) protocol is widely used to induce depressive-like behaviors in rodents, including anhedonia, weight changes, impaired self-care, and behavioral despair [18,19,20]. These models facilitate the identification of neurobiological substrates associated with the disorder and enable the development of improved pharmacological and therapeutic strategies for the diagnosis, monitoring, and treatment of depression.

In recent years, extracellular vesicles (EVs), particularly exosomes, have emerged as a promising approach for investigating cellular and molecular mechanisms underlying multiple pathological conditions [21,22,23]. EVs are nanosized lipid bilayer vesicles, typically ranging from approximately 30 to 200 nm in diameter, that mediate intercellular communication through the transfer of proteins, lipids, and nucleic acids [24]. Importantly, the molecular cargo of EVs reflects the physiological or pathological state of their cells of origin, suggesting that they may serve as informative indicators of disease processes [25]. In the central nervous system (CNS), EVs have been implicated in several critical physiological processes, including synaptic function and plasticity, neurogenesis, neuro-inflammation, and neural regeneration [26,27,28,29,30,31,32,33]. Furthermore, EVs can cross the blood–brain barrier (BBB), suggesting that EVs present in peripheral biofluids such as blood or cerebrospinal fluid (CSF) may reflect bidirectional communication between the CNS and peripheral tissues [22,27,34,35]. This property has generated increasing interest in EVs as a potential source of biomarkers for neurological and neuropsychiatric disorders [36,37], including depression. Indeed, several studies have reported alterations in EV characteristics in individuals with MDD. Analyses of morphological and physicochemical properties of plasma- or serum-derived EVs have revealed differences between patients with MDD and healthy controls [38,39]. In addition, multiple studies have focused on identifying differential EV cargo, particularly microRNAs (miRNAs), associated with depression. These include miR-139-5p [40], miR-207 [41], miR-26a [42,43], miR-494, miR-30c, miR-93, and miR-101 [43], as well as let-7e, miR-21-5p, miR-223, miR-145, miR-146a, and miR-155 [44,45], among others [46,47,48,49,50,51]. Several of these miRNAs have been investigated in vitro in neuronal, astrocytic, or microglial cultures [41,44,49,52,53], as well as in vivo in animal models [40,41,52,53], where they appear to regulate signaling pathways such as NF-κB, MAPK, Wnt, and mTOR, which are implicated in depressive-like behaviors [41,49,50,51].

Despite these advances, relatively few studies have investigated the protein cargo of EVs in the context of MDD, and comparative analyses between EVs derived from human patients and those obtained from experimental animal models remain limited. Understanding the similarities and differences between human and murine EV profiles may provide important insights into the translational relevance of rodent models for depression research. Therefore, in the present study, we compared the protein content of serum-derived EVs obtained from female patients diagnosed with MDD and from female mice subjected to CUMS, a well-established model of depressive-like behavior. By identifying common and differential EV protein signatures between humans and mice under healthy and depressive-like conditions, this study aims to evaluate EVs as a potential source of clinically relevant biochemical information and to explore their possible value as biomarker candidates for the diagnosis and monitoring of depression.

## 2. Materials and Methods

### 2.1. Participants

**Figure 1:**
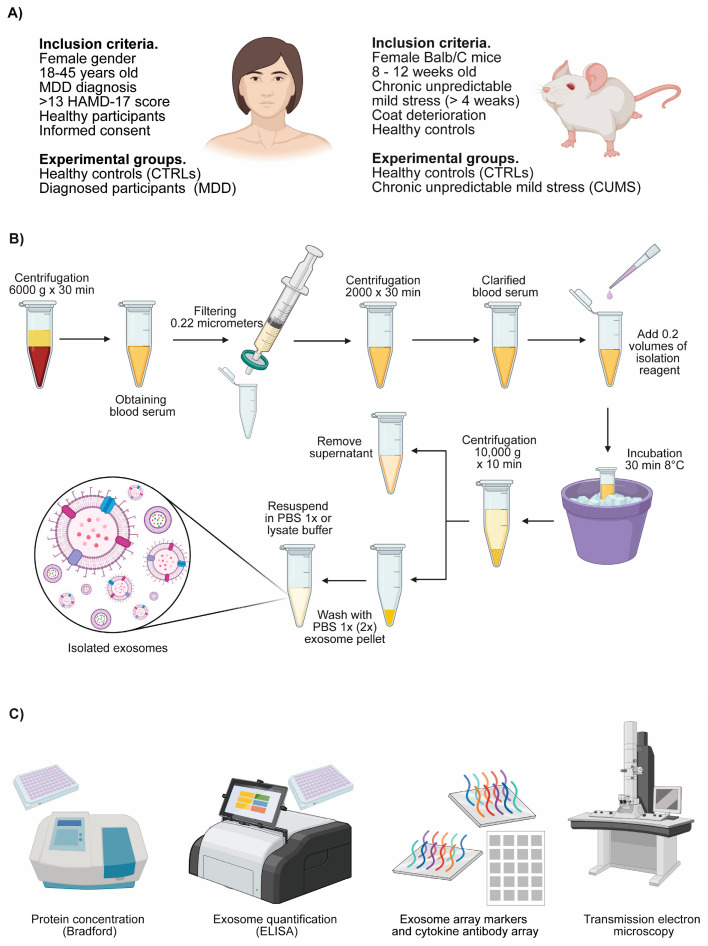
General diagram of the study. (**A**) Characterization of the human and mouse populations under study: inclusion criteria and experimental characteristics. (**B**) Collection, processing of human and murine blood serum samples, and isolation of exosomes using Total Exosome Isolation Reagent (from serum). (**C**) Techniques used for the analysis and characterization of exosomes isolated from blood serum: measurement of total protein concentration, quantification (number of isolated exosomes), determination of exosome protein content, and electron microscopy analysis (exosome ultrastructure).

Based on the fact that hormones influence neurotransmission, neuro-inflammatory responses, and HPA axis regulation [12,13], contributing to stress responses and vulnerability to depressive disorders in women [14], here, we focused on women. Thus, twenty women aged 22–36 years were enrolled in this study after providing written informed consent. Participants were recruited from the outpatient services of the Ramón de la Fuente Muñiz National Institute of Psychiatry (INPRFM). Twelve subjects were diagnosed with major depressive disorder (MDD) according to DSM-5 criteria by a certified psychiatrist and had a score greater than 13 on the 17-item Hamilton Depression Rating Scale (HDRS). Eight healthy control women (CTRL) were recruited from the general population and matched for age. Control participants were screened to exclude any current or past psychiatric disorders. All participants (diagnosed and healthy controls) were not receiving pharmacological treatment (including antidepressants, anti-inflammatory drugs, and antibiotics) at the time of enrollment. Subjects were excluded if they reported the use of hormonal or dietary supplements, contraceptives, or alternative treatments. Other exclusion criteria included the presence of infectious, inflammatory, metabolic, or autoimmune diseases, substance use (including alcohol, tobacco, and illicit drugs), and any history of psychotic, manic, or hypomanic episodes. Participants with known hormonal or gynecological disorders, including polycystic ovary syndrome or endometriosis, were excluded. No participants were pregnant during the study. Clinical, biochemical, and demographic variables were recorded (Table 1). The human study was conducted in accordance with the Declaration of Helsinki and approved by the Ethics Committee of the Instituto Nacional de Psiquiatría Ramón de la Fuente Muñiz (INPRFM; approval number CEI/C/002/2018, 15 January 2018) and by the Comisión Nacional de Bioética (CONBIOÉTICA, approval number CONBIOÉTICA-09-CEI-010-20170316, 15 January 2018) (Figure 1A).

### 2.2. Animals

To align our study performed in women to the general aim, we included female mice to be exposed to the CUMS. This model reflects clear effects of chronic stress on depression-like behavior and neurochemical modifications, as we mentioned in the last part of the Section 1. A total of twenty Balb/C female mice, 8–12 weeks old and weighing 20 ± 2 g, were used in this study and divided into two groups: ten subjects under the CUMS model and ten healthy control (CTRL) subjects. The animals were facilitated by the INPRFM, and they were held in standard laboratory plexiglass cages (28 × 17 × 15 cm) with a 12/12 h light/dark cycle (light on at 07:00 h and lights off at 19:00 h) and access to food and water ad libitum. All the handling procedures complied with the Official Mexican Standard for Animal Care, as well as international guidelines and protocols [54,55,56,57]. The animal study protocol was approved by the Institutional Animal Care and Use Committee of the INPRFM (approval number CICUAL/05/2022, 12 June 2024) (Figure 1A).

### 2.3. Induction and Evaluation of the Chronic Unpredictable Mild Stress Protocol

#### 2.3.1. Chronic Unpredictable Mild Stress Protocol and Hormone Quantification

**Figure 2:**
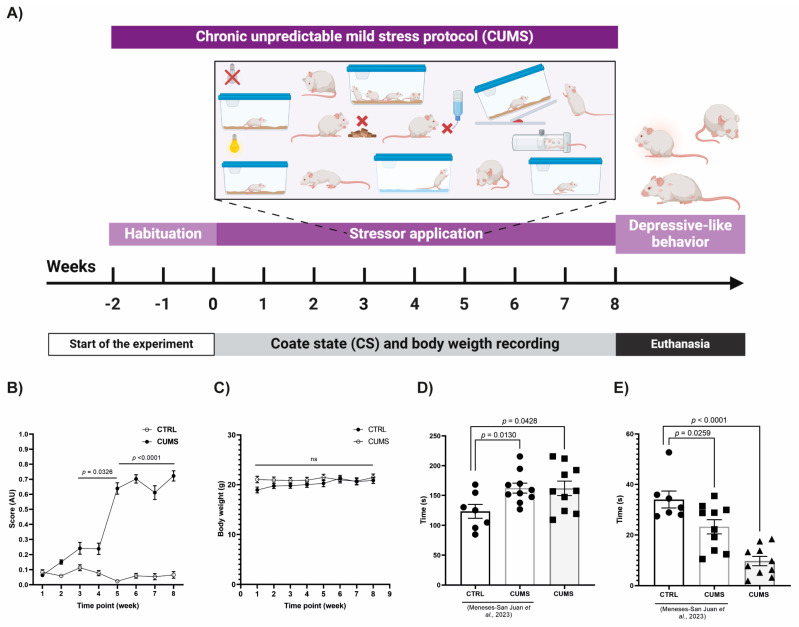
Implementation of the chronic unpredictable mild stress (CUMS) protocol to obtain murine exosome samples. (**A**) CUMS protocol overview: 2 weeks of habituation, followed by 1–8 weeks of stressor application and recording of coat state (CS) and body weight, ending with euthanasia in weeks 8–9. (**B**) Behavioral characterization: weekly CS and (weeks 3–5, *p* = 0.0326; weeks 5–8, *p* < 0.0001) (**C**) body weight were analyzed in control (CTRL) and stressed (CUMS) mice; no significant differences (ns) were observed between subjects exposed to the CUMS protocol and healthy CTRL subjects (*p* = 0.3182). Depressive-like behaviors emerged at weeks 4–5 of the protocol. Evaluation of the depressive-like behavior in mice under the CUMS protocol: (**D**) total immobility time significant increase in the immobility time (31.30%) compared to the previously reported control group (t = 2.213, df = 15; *p* = 0.0428) [64], and (**E**) immobility latency to the first immobility episode was significantly reduced in the CUMS-treated mice (71.42%) compared to the previously reported control group (t = 6.862, df = 15; *p* < 0.0001) [64]. At the end of the CUMS protocol, an increase in the manifestation of depressive-like behavior with total immobility time and a decrease in immobility latency to immobility are shown [64].

To induce depressive-like behaviors, the mice were subjected to the CUMS protocol. This protocol consisted of the application of one to three stressors of mild-to-moderate intensity during each day for a period of four to eight weeks (Figure 2A). The stressors were applied until the generation of observable and characteristic depressive-like behaviors in the mice (anhedonia, weight change, alterations in emotional self-care, psychomotor retardation, and hopelessness). The stressors included exposure to a cold room (4 °C), cage in horizontal rotation (40 rpm), inclined cage (45°), wet cage, empty cage, wet bedding (cage), overcrowding, light off (extension of the dark phase), light on (extension of the light phase), strobe light, predator odor, water deprivation, food deprivation, movement restriction, and reversal of light/dark cycle (Figure 2A). Also, in the CUMS-exposed group, the rodents underwent the forced swim test to confirm depressive like behavior [58,59,60]. According to Willner (2005) and Nollet (2021) [18,19], the sequence and timing of stressor application correspond to a logical and unpredictable order that does not harm the physical integrity of the animal according to the Grimace Scale [55,56,57]. The estrous cycle stage was determined by vaginal smear cytology according to Caligioni (2009) [61] at the end of the CUMS protocol and recorded for subsequent analysis. Also, corticosterone and 17β Estradiol were quantified by ELISA (Enzo Life Sciences, Farmingdale, NY, USA, ADI-900-097, ADI-900-008) according to the manufacturer’s instructions. The 96-well plates were read in a Promega ELISA reader (Glomax Discover, Madison, WI, USA Ref#GM3000) [61,62,63].

#### 2.3.2. Evaluation of the Coat State (CS) and Weight of Mice

Coat condition is described as one of the most replicable indexes or scales in the monitoring of self-care behavioral alterations induced by the CUMS model, validated as a useful parameter for studying depressive-like behaviors [65,66,67]. The coat state (CS) analysis consists of assigning a score from 0 to 1 to the coat appearance, where a value of 0 corresponds to a good coat condition (smooth, shiny and unaltered coat), a value of 0.5 corresponds to an intermediate condition (slightly fluffy coat), and a value of 1 corresponds to a very bad condition (fluffy and unkempt, with patches on the coat, almost all over the body) [18,19]. The recording of the score was performed in five areas of the mice’s body, namely, the head, neck, torso, abdomen, and tail, three times per week during the whole protocol, and the data obtained were analyzed to identify the appearance and evolution of depressive-like behaviors in the female Balb/C mice. Measurement of mouse body weight has also been reported as an important index of self-care behavioral alterations induced by the CUMS model. In this study, the weight of each mouse was recorded three times per week throughout the protocol, and statistical comparisons were made between experimental groups.

#### 2.3.3. Evaluation of the Depressive-like Behavior in Mice Exposed to the CUMS Protocol

The forced swim test (FST) was used to assess depressive-like behavior in the mice [58,59,68] exposed to the CUMS protocol, but not in the control group, to avoid confounding effects of the acute stress produced by the FST. The behavioral outcomes of the CUMS-exposed group were compared against previous data published by our group [64]. The test consisted of a single session performed at the end of the CUMS protocol. The mice were individually placed in a 12 × 30 cm cylinder filled to half its capacity with water maintained at room temperature (25 °C ± 2), resulting in a water depth of 15 cm, and allowed to swim for up to 5 min (300 s). During this period, the sessions were video-recorded and analysed using AnyMaze behavioural tracking software version 7.01 (Stoelting Co., Wood Dale, IL, USA) [64]. Immobility-related parameters, including the total immobility time and latency to immobility, were quantified. Immobility was defined as the absence of any movement for at least 2000 ms.

### 2.4. Serum Sample Collection and Processing

Human and murine blood and serum samples were processed according to the recommendations for collection, handling, storage, isolation, separation, characterization, and reproducibility described in the technical guidelines of the International Society for Extracellular Vesicles (ISEV), including MISEV2023 [69] and MiBlood-EV [70], among others [71,72,73]. In addition, the guidelines established in the Mexican Official Standards NOM-087 and NOM-166 were followed [74,75].

Fresh blood samples from the human participants were collected at the Clinical Laboratory of the INPRFM between 7:00 and 9:00 a.m. after at least 8 h of fasting. Blood was obtained from the antecubital vein by venipuncture using a BD Vacutainer^®^ system (21-gauge Eclipse needle and 5 mL SST tube for serum; BD Vacutainer, Becton Dickinson, Franklin Lakes, NJ, USA). Serum was separated by centrifugation at 1500× *g* for 15 min at room temperature (RT) using a Smart R17 centrifuge (Hanil Science Industrial, Gimpo, Republic of Korea) (Figure 1B). The mice were euthanized by decapitation, and fresh blood samples were collected in 1.5 mL microtubes. Brain tissue was also collected for subsequent histological analyses. Mouse serum was obtained after centrifugation at 6000× *g* for 30 min at RT using a MIKRO 200R centrifuge (Hettich Zentrifugen, Tuttlingen, Germany) (Figure 1B). All aliquots obtained from collected serum samples were processed within 2 h of blood collection. Serum samples were clarified to remove debris by filtration through a 0.22 μm nitrocellulose membrane followed by centrifugation at 2000× *g* for 30 min, prior to EVs isolation. Throughout the procedures, the samples were maintained under cold-chain conditions (4 °C ± 2). Unnecessary freeze–thaw cycles were avoided, and only three were allowed per sample. Finally, two to three microtubes containing aliquots of 0.1–0.5 mL were frozen and stored at −80 °C until further analysis, including the isolation and characterization of EVs (Figure 1B).

### 2.5. Isolation of Exosomes from Human and Murine Blood Serum

Extracellular vesicles were isolated using the Total Exosome Isolation reagent (from serum) (Invitrogen™, Thermo Fisher Scientific, Waltham, MA, USA, #4478360) according to the manufacturer’s protocol. Briefly, 500 μL of clarified serum obtained from human and mouse samples was mixed with 100 μL of the isolation reagent and incubated for 30 min at 8 °C. Exosomes were then precipitated by centrifugation at 10,000× *g* for 10 min using a Smart R17 centrifuge (Hanil Science Industrial, Gimpo, Republic of Korea). After removal of the supernatant, the pellet was washed twice with 1× PBS. This precipitation-based method allows the enrichment of exosome-associated EVs from serum samples. Finally, the EV pellets were resuspended in the appropriate buffer depending on the downstream application: 1× PBS for EV quantification, characterization, and morphological analyses (TEM), or 1× RIPA buffer for vesicle lysis and access to their molecular cargo (Figure 1B).

### 2.6. Characterization of Exosome-Enriched EVs from Human and Murine Blood Serum

#### 2.6.1. Total Protein Quantification and Exosome-Enriched EV Quantification

The total protein concentration of exosome-enriched EVs isolated from human and murine serum samples was determined using the Bradford assay (Abcam, Cambridge, UK, #AB119216). All samples were homogenized and lysed using 1× RIPA buffer supplemented with protease (cOmplete, Roche, Basel, Switzerland, #11697498001) and phosphatase inhibitors cocktail (PhosStop, Roche, #04906845001), and treated with three 15 s cycles of ultrasound (VibraCell™ SONICS, Newtown, CT, USA) at 4–8 °C. A standard curve was generated using bovine serum albumin (BSA; Research Organics, Cleveland, OH, USA, #1332A), and the total protein content in 1 μL of each sample was determined by measuring absorbance. Protein concentrations were calculated by linear regression analysis based on the BSA standard curve (Figure 1C).

In addition, the relative abundance of CD63-positive EVs was assessed using an Exo ELISA-Ultra CD63 assay (System Biosciences, Palo Alto, CA, USA, EXEL-ULTRA-CD63-1). Briefly, the equivalent of 100 μg of exosome-enriched EV-isolated protein from non-lysed exosome-enriched EVs preparations resuspended in PBS was incubated in a 96-well microplate with capture and binding buffer according to the manufacturer’s protocol. Subsequently, the wells were incubated with a primary antibody against the EV surface marker CD63 (a tetraspanin protein). Signal detection was performed using a colorimetric substrate, and absorbance was measured at 450 nm using a Glomax Discover multimode plate reader (Promega, Madison, WI, USA, Ref#GM3000). Quantification was performed using a CD63 standard curve followed by linear regression analysis of absorbance values for each sample (Figure 1C). Finally, the particle-to-protein ratio—defined as the ratio of total protein concentration to the number of particles—was calculated for the experimental groups of both species (CTRL and MDD; CTRL and CUMS).

#### 2.6.2. Exosome-Enriched EV Array Markers and CD63 Slot Blot Analysis

**Figure 3:**
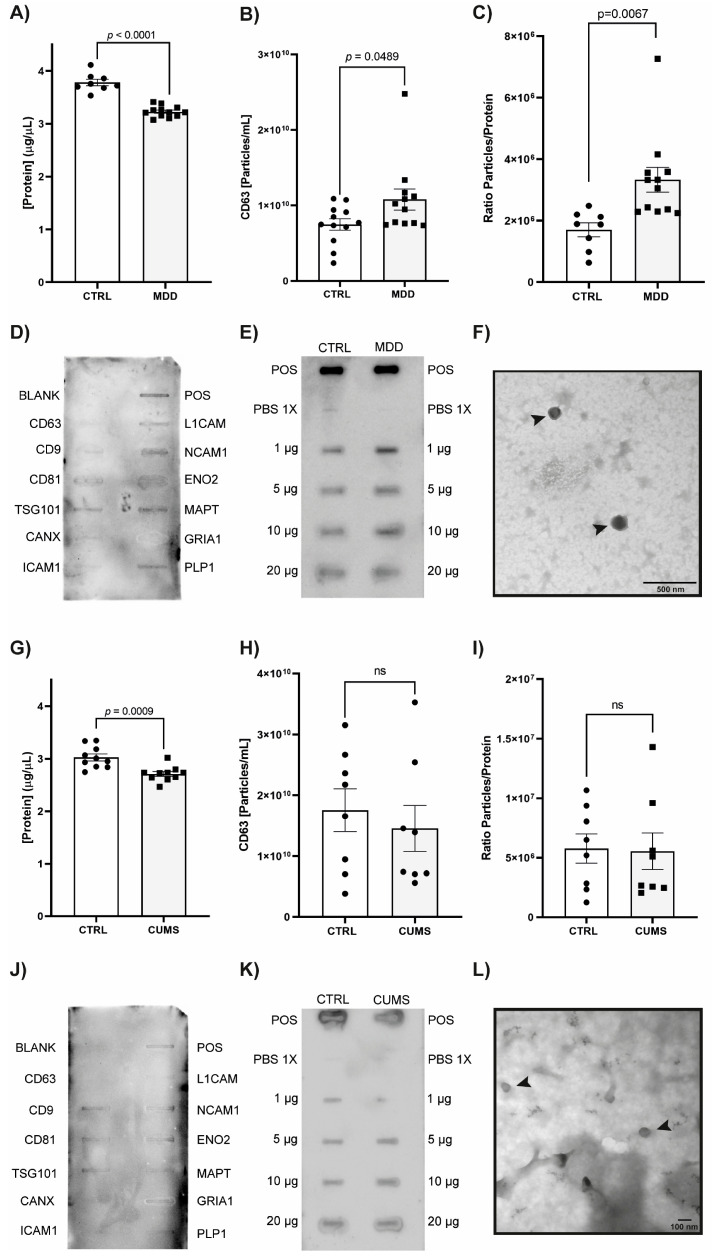
Characterization of human and murine exosomes isolated from blood serum. (**A**,**G**) Identification of transmembrane markers commonly associated with exosomes, as well as markers of glial and neural cells. (**B**,**H**) Slot blot of human and murine exosomes: comparison of 1, 5, 10, and 15 μg of total protein from non-lysed exosome-enriched EVs derived from human (CTRL, MDD) and murine (CTRL, CUMS) blood serum. PBS 1× as negative spot, and antibody CD63 as positive spot. (**C**,**I**) Micrographs of human and murine exosomes, by transmission electron microscopy. Arrowheads indicate extracellular vesicles isolated from blood serum samples. (**D**,**J**) Determination of total protein concentration (μg/μL) in human and murine exosomes. Significant decreases of 14.62% (*p* < 0.0001) and 10.40% (*p* = 0.0009) were observed in the protein concentration in the MDD and CUMS groups vs. the human and murine CTRL groups, respectively. (**E**,**K**) Relative abundance of exosome particles (particles/mL) in human and murine samples. Only a significant increase of 44.30% in the relative abundance of exosome particles was identified for the MDD group (*p* = 0.0489), no significant differences (ns) were observed in murine samples (*p* = 0.5393) (**F**,**L**) Ratio of relative abundance of CD63-positive particles/protein, the analysis showed only a significant differences between the CTRL and MDD groups (*p* = 0.0067), but not significant differences (ns) in the murine samples (*p* = 0.9057).

Analysis of EV-associated transmembrane markers was performed using an Exo-Check Antibody Array (Neuro) Standard Kit (System Biosciences, EXORAY500A-8, Palo Alto, CA, USA), following the manufacturer’s instructions. Briefly, 200 μg of exosome-enriched EVs resuspended in 1× PBS were lysed using 10× lysis buffer to obtain a final concentration of 1×. The lysed exosome-enriched EV samples from one human or one mouse were then incubated with the antibody array membrane containing immobilized antibodies against common EV markers overnight at 4–8 °C. Chemiluminescent HRP substrate (Immobilon^®^ Western, Cat. No. WBKLS0100, Millipore™, Burlington, MA, USA) was added, and signals were detected using a ChemiDoc Touch Imaging System (Bio-Rad, Hercules, CA, USA) (Figure 1C and Figure 3D,J).

The presence of the EV-associated tetraspanin CD63 was confirmed by slot blot analysis. Briefly, 1, 5, 10, and 15 μg of total protein from non-lysed exosome-enriched EVs derived from human (CTRL, MDD) and murine (CTRL, CUMS) blood serum samples were loaded onto a nitrocellulose membrane (45 μm size pore, Millipore™ Cat. No. HATF00010, Dublin, Ireland) using a vacuum-driven slot blot manifold (Hybri-Slot™ Manifold, Cat. No. 1052M, Life Technologies, Inc., Waltham, MA, USA). Phosphate-buffered saline (PBS 1×) was used as a negative control, and primary antibody CD63 was used as a positive control. After the sample application, the membrane was air-dried at room temperature and subsequently blocked with a solution containing 3% non-fat dry milk, 0.1% bovine serum albumin (BSA), and 0.1% Tween-20 in Tris-buffered saline (TBS) for 30 min under agitation. The membrane was then washed three times for 10 min with TBS-T (0.05% Tween-20) under agitation. Then, the membrane was incubated overnight at 4–8 °C under agitation with a primary antibody against CD63 (1:2000 dilution in TBS-T 0.05%; Ab216130, Abcam, Cambridge, UK). After washing, the membrane was incubated with an HRP-conjugated anti-rabbit secondary antibody (1:10,000 dilution in TBS-T 0.05%) for 1 h at room temperature under agitation. After the incubation, the membrane was washed three times with TBS-T. Chemiluminescent detection was performed using an HRP substrate (Immobilon^®^ Western, Cat. No. WBKLS0100, Millipore™, Burlington, MA, USA). Signals were acquired with a ChemiDoc Touch Imaging System (Bio-Rad), and densitometry was done with Imagelab software Version 6.1.0 build 7 (Bio-Rad, Hercules, CA, USA) (Figure 1C and Figure 3E,J).

#### 2.6.3. Transmission Electron Microscopy Analysis

The morphology and size of isolated exosome-enriched EVs were evaluated by transmission electron microscopy (TEM). Briefly, a 200-mesh copper grid coated with carbon film (Electron Microscopy Sciences, Morgantown, PA, USA) was placed on a drop of exosome-enriched EV suspension resuspended in 1× PBS and incubated for adsorption. Excess liquid was removed using Whatman filter paper, and the grids were fixed with 2.5% glutaraldehyde. The grids were then washed twice with filtered deionized water (0.22 μm) and negatively stained with uranyl acetate. Excess stain was removed using filter paper, and the grids were allowed to air-dry. The samples were examined at accelerating voltages of 60–100 kV using a JEOL JEM-1010 transmission electron microscope (JEOL, Tokyo, Japan) equipped with an ATM digital camera (JEOL, Tokyo, Japan) (Figure 1C).

### 2.7. Analysis of Cytokine Cargo in Serum-Derived Exosome-Enriched EVs

#### 2.7.1. Human and Mouse Cytokine Array Analysis

**Figure 4:**
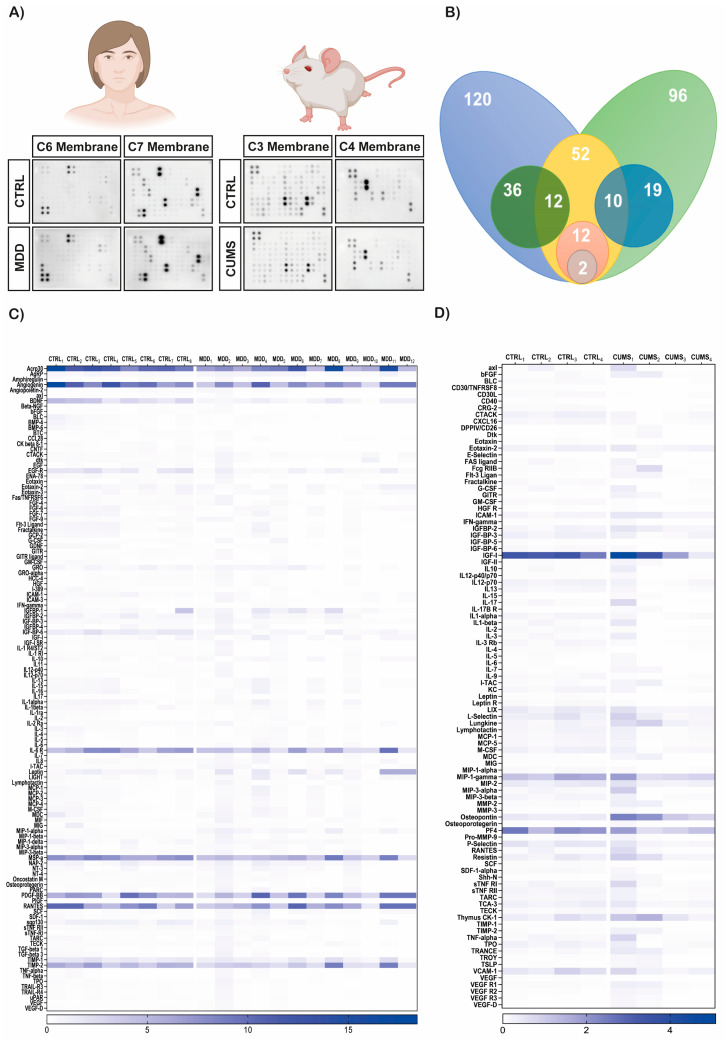
Semi-quantitative analysis of human and murine exosome content: antibody microarrays for cytokines/chemokines. (**A**) Representative images of C6 and C7 membrane arrays for the analysis of 120 human proteins and C3 and C4 membranes for the analysis of 96 murine proteins, CTRL groups (both species), and MDD and CUMS groups. (**B**) Venn diagram, comparison of human and murine cytokine/chemokine profiles in microarray membranes for each group. A total of 52 homologous proteins were identified between the membranes of each species. Statistical analyses showed 12 and 10 significant proteins in the homologous protein set between humans and mice, two of which are common to both species (BLC, Fractalkine). (**C**,**D**) Heat maps of the optical density analysis of the expression of the 120 and 96 human and murine proteins, respectively. The expression of the protein profile (row) for each of the experimental subjects (column) is shown.

A semi-quantitative analysis of cytokine and chemokine expression was performed using lysed exosome-enriched EV samples isolated from human and mouse serum samples obtained from the experimental groups (Figure 4A). All the human samples were analyzed (Figure 4C), but only eight mouse samples (n = 4 CTRL and n = 4 CUMS) were analyzed due to the quantity of protein available (Figure 4D). Cytokine and chemokine profiles were evaluated using Human Cytokine Antibody Array C1000 (AAH-CYT-1000-8) and Mouse Cytokine Antibody Array C1000 (AAM-CYT-1000-8) kits (RayBiotech^®^, C-Series, Norcross, GA, USA), allowing the simultaneous detection of 120 and 96 analytes, respectively. Both assays were performed in a two-membrane format (C6 and C7 for human samples; C3 and C4 for mouse samples) according to the manufacturer’s instructions. The arrays are based on sandwich immunoassay chemistry with chemiluminescent detection and exhibit detection sensitivities in the pg/mL range (Figure 4A). For each membrane, 250 µg of total EV protein was used for incubation.

Chemiluminescent detection was performed using an HRP-based substrate (Immobilon^®^ Western, Cat. No. WBKLS0100, Millipore™, Burlington, MA, USA), and membrane signals were acquired using a ChemiDoc™ Touch Imaging System (Bio-Rad Laboratories, Hercules, CA, USA). Signal intensities were quantified by densitometric analysis using Image Lab 6.0.1 software (Bio-Rad Laboratories, Hercules, CA, USA). Spot intensities were measured as integrated optical density (OD) using identical circular regions of interest for all spots, and duplicate spots corresponding to each cytokine were averaged. Background correction and signal normalization were performed using the manufacturer’s analysis templates (RayBiotech^®^, C-Series Cytokine Array Analysis Tool, Norcross, GA, USA). Normalized signal intensities were calculated relative to the mean signal of the internal positive and negative control spots present on each membrane. These normalized values were subsequently used to calculate relative cytokine expression levels between the experimental groups. For the statistical analysis of the human and mouse cytokine arrays, unpaired Student’s *t*-tests were performed to evaluate the relative expression levels of each cytokine and chemokine (120 and 96 analytes for the human and mouse arrays, respectively). In addition, a two-way ANOVA (with Bonferroni’s test for multiple comparisons) was conducted to analyze the relative expression of the 52 cytokines and chemokines shared between both membranes, considering species and experimental condition as factors (Figure 4B).

#### 2.7.2. Bioinformatics and Proteomic Data Analysis of Exosome-Enriched EV Cargo

Bioinformatic analyses were performed based on the statistical results obtained from the comparisons of relative cytokine and chemokine expression levels between the experimental groups. Only proteins showing statistically significant differences between groups and species were included in the analyses (Figure 4B). Protein annotation and functional information were obtained using the UniProt database (Universal Protein Resource, version release 2024_01. Developed and maintained by the UniProt Consortium, including the European Bioinformatics Institute (EMBL-EBI, Hinxton, Cambridge, UK), the Swiss Institute of Bioinformatics (SIB, Geneva, Switzerland), and the Protein Information Resource (PIR, Washington, DC, USA); Access on 24 November 2024, in https://www.uniprot.org/). Protein–protein interaction (PPI) networks were analyzed using the STRING database platform (Functional Protein Association Networks, STRING Consortium, version 11.5. Developed and maintained by the STRING Consortium, including the Swiss Institute of Bioinformatics (SIB, Geneva, Switzerland), the Novo Nordisk Foundation Center for Protein Research (CPR, University of Copenhagen, Copenhagen, Denmark), and the European Molecular Biology Laboratory (EMBL, Heidelberg, Germany); Access on 24 November 2024, in https://string-db.org/) to identify functional interaction networks between proteins and their associated genes. Network construction was performed using a minimum required interaction score of 0.700 (high confidence) [76,77]. Functional enrichment analyses were subsequently carried out to evaluate associated biological processes, cellular components, and molecular functions using Gene Ontology (GO) annotation through the WebGestalt platform (WEB-based Gene Set Analysis Toolkit, Version 2024, Houston, TX, USA; Access on 24 November 2024, in http://www.webgestalt.org/). These analyses were complemented by pathway enrichment analysis using the KEGG (Kyoto Encyclopedia of Genes and Genomes) database [78,79]. In summary, an over-representation analysis (ORA) was performed separately for each organism (*Homo sapiens* and *Mus musculus*). The parameters for the enrichment analysis included a minimum of 5 IDs and a maximum of 2000. Multiple testing correction was applied using the Benjamini–Hochberg false discovery rate (FDR) method, and pathways with an FDR-adjusted *p*-value < 0.05 were considered statistically significant (q-value). All the bioinformatic analyses were performed in November 2024.

### 2.8. Statistical Analysis

Statistical analyses were performed using Microsoft Excel version 2603 (Microsoft Corporation, Redmond, WA, USA), SigmaPlot version 15.0 (Systat Software, Inc., San Jose, CA, USA) and GraphPad Prism version 8.4.2 (GraphPad Software, Inc., Boston, MA, USA). Data are presented as mean ± standard error of the mean (SEM). The appropriate statistical test was selected for each determination. Data distribution was assessed for normality using the Shapiro–Wilk test. Comparisons between two groups were performed using an unpaired two-tailed Student’s *t*-test for normally distributed data or the Mann–Whitney U test for non-normally distributed data. Multiple comparison corrections were applied using the Benjamini–Hochberg procedure. Comparisons involving two or more groups and factors were analyzed using two-way analysis of variance (ANOVA), followed by Bonferroni’s post hoc test for multiple comparisons. Differences were considered statistically significant at *p* < 0.05.

## 3. Results

### 3.1. Clinical and Biochemical Differences Are Notorious Between Healthy Controls and MDD Patients and Hormone Quantifications in CUMS-Exposed Mice

Table 1 shows the results of the Student’s unpaired two-tailed *t*-test for the 20 clinical, biochemical and hormonal parameters analyzed. For the menstrual cycle parameters, the Mann–Whitney U test was used for day of the cycle, and Fisher’s exact test was used for phase of the cycle. All healthy controls and depressed female participants were of the same age range and, according to their clinical histories and the inclusion and exclusion criteria, did not manifest any other medical or psychiatric disorder during their participation in the study.

There was a significant difference between the healthy control group (2.41 ± 2.15) and participants with MDD (23.92 ± 4.68) on the 17-HDRS scale (*p* = 0.0001), indicating that participants with MDD had experienced symptoms for more than 2 weeks, as defined by the DSM-5. In the hormonal evaluations, only FSH levels differed significantly (*p* = 0.0328) between healthy control subjects (3.96 ± 1.76) and participants with MDD (5.66 ± 1.78). On the day of the menstrual cycle when blood was collected, there was a significant difference between the healthy control group (19.43 ± 3.631) and the participants with MDD (9.556 ± 2.561). Interestingly, although there were no significant differences in the menstrual cycle phase between the groups (*p* = 0.0623), there was a possible trend toward a predominance of the luteal phase in the CTRL group (n = 5; 62.50%) and of the follicular phase in the MDD group (n = 10; 83.33%).

Table 2 shows the results of the Student’s unpaired two-tailed *t*-test for the quantification of corticosterone and 17-β Estradiol. For the estrous cycle parameters, the Mann–Whitney U test was used for day of the cycle, and Fisher’s exact test was used for phase of the cycle. Here, we did not find significant differences in corticosterone or 17-β Estradiol. The results suggest a slight increase in corticosterone in the CUMS-exposed mice compared with the control group. Contrary results were found after the quantification of 17-β Estradiol, with a slight decrease in the CUMS-exposed mice. However, in both cases we did not find a statistically significant difference.

### 3.2. Chronically Stressed Mice Show Alteration in Coat State Without Modification to Body Weight

The weekly analysis of CS in the CTRL and CUMS groups is shown in Figure 2B. A two-way ANOVA of repeated measures was performed to compare the effect of stress exposure on coat condition (CS) and the time (weeks) at which changes in this behavior were observed. For the time factor, it was identified that the period between weeks 4 and 5 in the group exposed to the CUMS protocol was when a generalized poor condition and significant deterioration of the coat began to be observed (F(2.358, 28.29) = 52.70; *p* < 0.0001), with an increase in the mean CS score, characteristic of the manifestation of depressive-like behavior, compared to the CTRL group, which was not exposed to the CUMS protocol (Figure 2B). Regarding the stress exposure factor, a significant difference was observed between the CTRL and CUMS groups in the CS (F(1, 12) = 469.3; *p* < 0.0001) (Figure 2B). Finally, a significant interaction between the two factors (stress exposure and time) was identified (F(7, 84) = 65.57; *p* < 0.0001). On the other hand, in the weekly body weight records for each subject between groups (Figure 2C), no significant differences were observed between subjects exposed to the CUMS protocol and healthy CTRL subjects (F(1, 39) = 1.022; *p* = 0.3182). Taken together, these results suggest that mice under chronic stress showed impaired grooming, indicative of impaired self-care, without differences in body weight.

### 3.3. Chronically Stressed Mice Show Depressive-like Behaviors

As part of the behavioral characterization, at the end of the CUMS protocol, the rodents were exposed to acute stress induced by FS. Thus, the outcomes of those rodents were compared with data previously generated [68]. The mice subjected to the CUMS protocol exhibited a significant increase in the immobility time (31.30%) compared to the previously reported control group (t = 2.213, df = 15; *p* = 0.0428) [68] (Figure 2D). Similarly, latency to the first immobility episode was significantly reduced in the CUMS-treated mice (71.42%) compared to the previously reported control group (t = 6.862, df = 15; *p* < 0.0001) [68] (Figure 2E). Taken together, these findings are consistent with a depressive-like state in mice and in agreement with the CS measurements for the CUMS protocol (Figure 2B).

### 3.4. Exosome-Enriched EV Array Markers, CD63 Slot Blot and Transmission Electron Microscopy Analysis

Also, we analyzed the presence of exosome-enriched EV markers in one human and one mouse sample, respectively. With this approximation, we wanted to validate that the precipitation-based method enriches exosome-associated EVs from serum samples. From this approximation, we visually identified the presence of tetraspanin 29 (CD9), tetraspanin 28 (CD81), and the tumor susceptibility gene 101 (TSG101) in the human and murine samples; these are commonly used as exosome markers for their characterization (Figure 3A,G). Furthermore, in the exosome protein marker array, calnexin (CANX) and the intracellular adhesion molecule 1 (ICAM1) were barely visible or undetected. The low abundance or absence of CANX, a control for cellular contamination, suggests that EVs were correctly and effectively isolated using this precipitation-based method; the absence of ICAM1 may suggest low enrichment of EVs with this general EV marker. In addition, we identified the presence of proteins related, but not exclusively, to neural lineage, such as L1 transmembrane protein (L1CAM), neural cell adhesion molecule 1 (NCAM1), enolase 2 (ENO2), and total tau protein (MAPT), and proteins possibly only linked to oligodendrocyte lineage, such as proteolipid protein 1 (PLP), for human exosome-enriched EVs. In the murine exosome-enriched EV samples, we detected low immunoreactivity of NCAM, ENO2, MAPT, and glutamate receptor 1 (GRIA1). However, tetraspanin 30 (CD63) was not correctly detected. Thus, we performed slot blots with exosome-enriched EVs from human CTRL and MDD samples (Figure 3B) and murine CTRL and CUMS samples (Figure 3H, Supplementary Figure S1). We identified CD63 in both species, suggesting that the precipitated samples contain this exosomal marker.

Moreover, TEM analysis revealed the presence of small EVs, ranging from 30 to 180 nm, in both the human and murine samples (Figure 3C,I). In the negative staining method, these EVs corresponded to the size described for small EV-like exosomes. The exosome-enriched EVs of both species were similar. Taken together, these results suggest that the isolated EVs are exosome-enriched EVs.

### 3.5. Exosome-Enriched EVs of Humans and Mice: Determination of Total Protein and Quantification

Quantification of total protein from exosome-enriched EVs isolated from both human groups (CTRL and MDD) and murine blood serum (CTRL and CUMS) was performed for each experimental group (Figure 3D,J). We found a 14.62% significant decrease in protein content in the MDD group compared to the CTRL group (3.782 μg/μL ± 0.06120 vs. 3.229 μg/μL ± 0.02994; t = 8.975, df = 18, *p* < 0.0001, respectively; Figure 3D). In the murine exosome-enriched EVs samples, a 10.40% significant decrease in protein content was also found for the group of mice exposed to the CUMS protocol compared to the CTRL mice (2.712 μg/μL ± 0.04521 vs. 3.027 μg/μL ± 0.06536; t = 3.962, df = 18, *p* = 0.0009, respectively; Figure 3J).

In addition, the relative abundance of CD63-positive EVs was determined by ELISA from both human groups (CTRL and MDD) and murine blood serum (CTRL and CUMS) samples of isolated exosome-enriched EVs (Figure 3E,K). For the human samples, a 44.30% significant increase in the relative abundance of CD63-positive EVs (particles/mL) was identified in the MDD group compared to the CTRL (t = 2.085, df = 22, *p* = 0.0489, mean ± SEM, 1.07 × 10^10^ particles/mL ± 1.39 × 10^9^ vs. 7.47 × 10^9^ particles/mL ± 7.55 × 10^8^; respectively; Figure 3E), and in the murine samples, no significant differences were found between the CTRL and CUMS groups (t = 0.5693, df = 14, *p* = 0.5693; mean ± SEM, 1.75 × 10^10^ particles/mL ± 3.52 × 10^9^ vs. 1.45 × 10^10^ particles/mL ± 3.75 × 10^9^; respectively; Figure 3K).

Finally, analysis of the relative abundance of the CD63-positive EVs/total protein ratio showed significant differences between the CTRL and MDD groups (t = 3.066, df = 18, *p* = 0.0067; mean ± SEM, 1,701,481 particles/protein ± 224,742 vs. 3,325,148 particles/protein ± 402,686; respectively; Figure 3F), but not in the murine samples (t = 0.1206, df = 14, *p* = 0.9057; mean ± SEM, 5,776,901 particles/protein ± 1,224,055 vs. 5,539,874 particles/protein ± 1,537,386; respectively; Figure 3L). In the case of the CTRL and MDD groups, an inverse result was seen when we analyzed the total protein/relative abundance of CD63-positive EVs (t = 3.139; df = 18, *p* = 0.0057; mean ± SEM, 7.115 × 10^−7^ protein/particles ± 1.428 × 10^−7^ vs. 3.348 × 10^−7^ protein/particles ± 2.785 × 10^−8^, respectively; Supplementary Figure S2).

### 3.6. Proteomic and Bioinformatic Analysis of Protein Content of Human and Murine Exosomes

Optical density analysis of 120 human and 96 murine proteins (Figure 4A,C,D) revealed differential expression patterns between the experimental groups. In the human samples, 36 proteins showed statistically significant differences between the CTRL and MDD groups (Table 3, Figure 4B). In the murine dataset, 19 proteins were significantly differentially expressed between the CTRL and CUMS groups (Table 4, Figure 4B).

**Figure 5:**
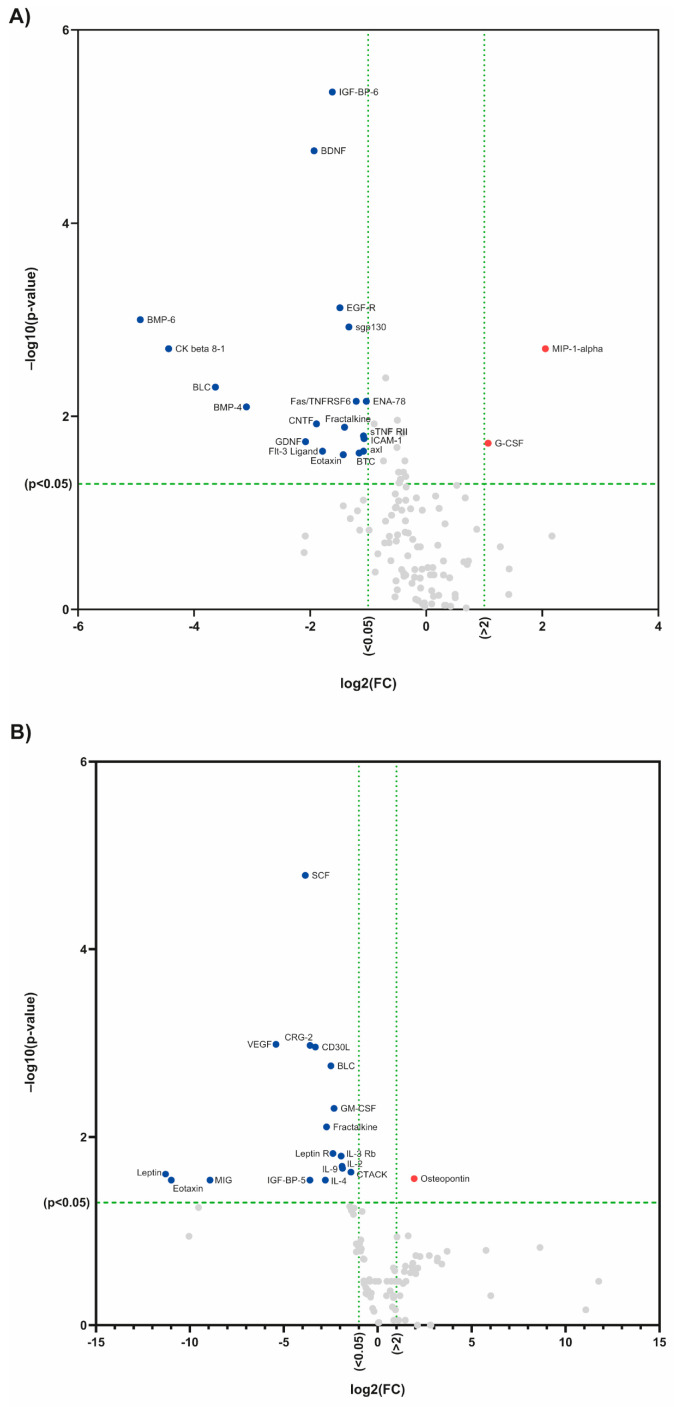
Volcano plot analysis of cytokine and chemokine expression in human and murine samples. (**A**) Expression profile of the 120 human cytokines and chemokines analyzed. (**B**) Expression profile of the 96 murine cytokine and chemokine panel analyzed. Blue dots represent significantly downregulated cytokines and chemokines, whereas red dots indicate significantly upregulated cytokines and chemokines. Gray dots represent non-significant cytokines and chemokines. Thresholds for statistical significance and fold change are indicated by green dashed lines.

To complement the findings derived from the sets of significantly altered proteins across the human and murine datasets, volcano plots were generated as a graphical representation integrating fold change (MDD/CTRL and CUMS/CTRL) and statistical significance (−log10 *p*-value) to identify differentially expressed proteins across experimental conditions (Figure 5). This approach enables rapid visualization of proteins exhibiting both biologically relevant changes and statistical significance, thereby facilitating the identification of potential molecular candidates (Table 3, Table 4 and Table 5). Nineteen proteins were identified as significantly downregulated in the human dataset, including IGF-BP-6, BDNF, BMP-6, EGF-R, CK-beta-8-1, sgp130, BLC, BMP4, FAS/TNFRSF6, ENA-78, CNTF, fractalkine, GDNF, sTNFRII, ICAM-1, Flt-3 ligand, eotaxin, Axl, and BTC, while two proteins (MIP-1α and G-CSF) were significantly upregulated (Figure 5A). In the murine dataset (Figure 5B), seventeen proteins were found to be significantly downregulated, including SCF, CRG-2, VEGF, CD30L, BLC, GM-CSF, fractalkine, leptin receptor, IL-13Rβ, IL-2, IL-9, IL-4, CTACK, IGF-BP-5, MIG, eotaxin, and leptin, whereas only one protein, osteopontin, was significantly upregulated. All significantly altered proteins identified in both datasets were part of the original sets of differentially expressed proteins determined for each species (Table 3 and Table 4).

Comparative analysis of the 52 proteins shared between the human and murine arrays identified 12 proteins with significant interspecies differences (Table 5, Figure 4B). Among these, 12 and 10 proteins were differentially expressed in humans and mice, respectively (Figure 4B). Notably, B lymphocyte chemoattractant (BLC), fractalkine (CX3CL1) and IGFBP6, among others, were significantly altered in both species (Table 3 and Table 4), showing downregulated expression in both the MDD patients and CUMS-exposed mice (Figure 5). These findings suggest a possible conserved role for EV-associated proteins in the context of major depressive disorder and depressive-like behavior.

**Figure 6:**
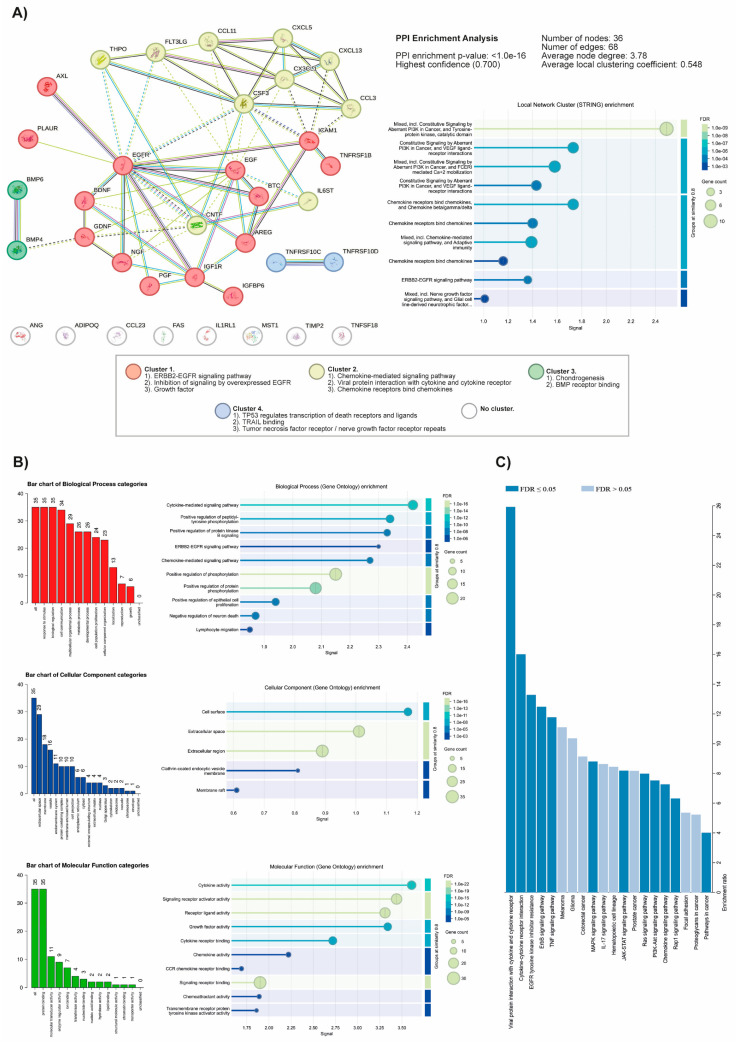
Proteomic and bioinformatic analysis of thirty-six human proteins. (**A**) Protein–protein interaction (PPI) analysis: *p*-value < 1.0 × 10^−16^, 36 nodes, four clusters. Cluster 1: red, fourteen proteins; cluster 2: gray, ten proteins; cluster 3: two proteins; cluster 4: two proteins; and a no-cluster set of eight proteins. All edges represent functional associations or physical interactions among proteins. Edge thickness reflects interaction confidence, whereas edge color indicates the evidence source used for association prediction: red, gene fusion; green, gene neighborhood; blue, gene co-occurrence; purple, experimental evidence; yellow, text mining; light blue, curated databases; and black, co-expression. Dashed lines indicate interactions connecting distinct protein clusters identified within the network. (**B**) Gene ontology analysis of biological processes (red bar chart) showing association in signaling pathways, including cytokine-mediated signaling, positive regulation of protein kinase B, protein phosphorylation, and negative regulation of neuron death. Cellular components (blue bar chart) are mainly associated with cell surface, space, and extracellular region pathways. Molecular functions (green bar chart) are associated with cytokine, chemokine, growth factor activity, and cytokine receptor binding. (**C**) Analysis of signaling pathways (KEGG) associated with the set of 36 proteins.

**Figure 7:**
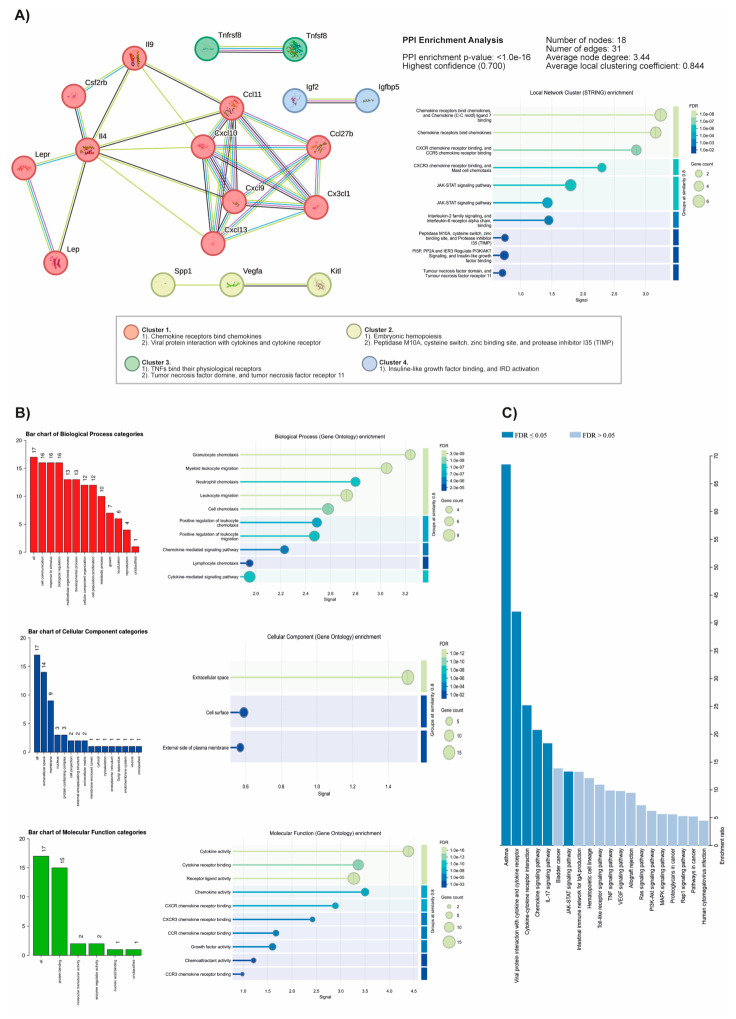
Proteomic and bioinformatic analysis of nineteen murine proteins. (**A**) Protein–protein interaction (PPI) analysis showing a *p*-value of <1.0 × 10^−16^, with 18 nodes and four clusters. Cluster 1 contains a red set of eleven proteins; cluster 2 has a gray set of three proteins; clusters 3 and 4 each have two proteins. All edges represent functional associations or physical interactions among proteins. Edge thickness reflects interaction confidence, whereas edge color indicates the evidence source used for association prediction: red, gene fusion; green, gene neighborhood; blue, gene co-occurrence; purple, experimental evidence; yellow, text mining; light blue, curated databases; and black, co-expression. Dashed lines indicate interactions connecting distinct protein clusters identified within the network. (**B**) Gene ontology analysis of biological processes (red bar chart) showing associations with signaling pathways, mainly chemotaxis (granulocytes, neutrophil, leukocyte, lymphocyte), as well as chemokine-mediated and cytokine-mediated signaling. Cellular components (blue bar chart) are mainly associated with the cell surface, extracellular space, and outer plasma membrane. Molecular functions (green bar chart) indicate associations with cytokine and chemokine activity, cytokine receptor binding, and chemokine receptor binding (CXCR, CXCR3, CCR, CCR3). (**C**) Analysis of signaling pathways (KEGG) associated with the set of 18 proteins.

**Figure 8:**
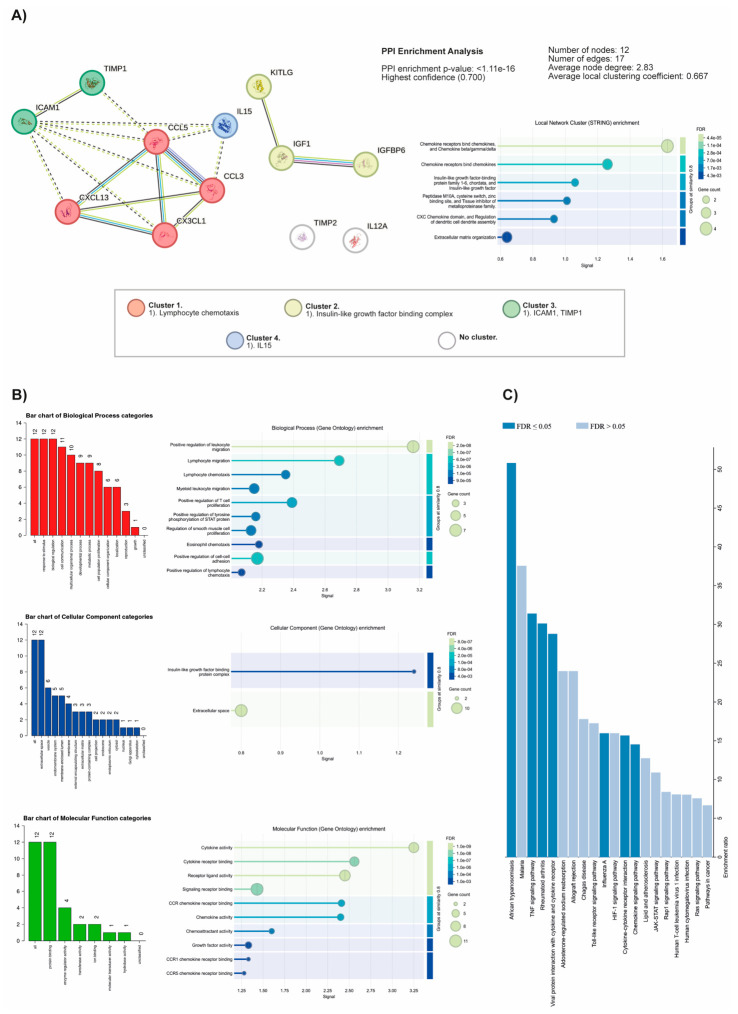
Proteomic and bioinformatic analysis of the set of twelve human and murine proteins. (**A**) Protein–protein interaction (PPI) analysis; *p*-value: <1.11 × 10^−16^; 12 nodes and four clusters. Cluster (1), set of four proteins; cluster (2), gray set of three proteins; cluster (3), two proteins; cluster (4), one protein. All edges represent functional associations or physical interactions among proteins. Edge thickness reflects interaction confidence, whereas edge color indicates the evidence source used for association prediction: red, gene fusion; green, gene neighborhood; blue, gene co-occurrence; purple, experimental evidence; yellow, text mining; light blue, curated databases; and black, co-expression. Dashed lines indicate interactions connecting distinct protein clusters identified within the network. (**B**) Gene ontology analysis of biological processes (red bar chart) with association in signaling pathways: regulation of leukocyte migration, lymphocyte migration and chemotaxis, regulation of T cell proliferation, regulation of tyrosine phosphorylation of STAT protein, and regulation of cell–cell adhesion, mainly; cellular components (blue bar chart) related to insulin-like growth factor binding protein complex, and extracellular space; molecular functions (green bar chart) with association in signaling pathways: cytokine and chemokine activity, cytokine CCR chemokine, CCR1 and CCR5 chemokine receptor binding, chemoattractant and growth factor activity, mainly. (**C**) Analysis of signaling pathways (KEGG) associated with the set of 12 proteins.

To explore the biological relevance of these alterations, protein–protein interaction networks were analyzed using the STRING database. Functional enrichment analyses were performed using Gene Ontology (GO) and KEGG pathway annotations via WebGestalt. This integrative approach enabled the identification of enriched biological pathways, molecular functions, and interaction networks associated with the cytokine cargo of serum-derived exosome-enriched EVs from the healthy control participants and individuals with MDD, as well as from the control mice and the mice exhibiting depressive-like behavior following exposure to the CUMS protocol. Interspecies comparisons further revealed both shared and distinct EV-associated cytokine signatures between human and murine conditions (Figure 6, Figure 7 and Figure 8; Table 3, Table 4 and Table 5).

### 3.7. The Human Protein Cluster Shows a Relationship Between ERBB2/EGFR and Chemokine-Mediated Signaling Pathways

PPI analysis of the set of 36 human exosome proteins (PPI enrichment *p*-value: <1.0 × 10^−16^; 0. 700 highest confidence value) and clustering (Figure 6A, Table 6) identified two subsets or clusters of proteins associated with different signaling pathways, where cluster 1 (red) and cluster 2 (green) integrated 14 proteins in the ERBB2-EGFR signaling pathway, growth factor, and inhibition of signaling by overexpressed EGFR pathways and, on the other hand, 10 proteins in the chemokine-mediated signaling pathway, viral protein interaction with cytokines and cytokine receptor and chemokine receptor-bound chemokines, respectively. Interestingly, significant protein–protein interaction between both clusters was identified: GDNF, EGF, EGFR, ICAM-1, and NGF in cluster 1, and CCL11, CCL3, CNTF, CX3CL1, CXCL13, CSF3, FLT3LG, IL6ST, and THPO in cluster 2 (dotted line, Figure 5A). This also allowed us to associate them with other processes and signaling pathways located in the cluster network (graph Local network, STRING enrichment, Figure 6A).

In addition, GO analysis of the associated biological processes identified the participation of this set of proteins in the categories of stimulus response, biological regulation, and cellular communication for the cytokine-mediated signaling pathway, positive regulation of protein kinase B signaling, ERBB2-EGFR signaling pathway, and chemokine-mediated signaling pathway, mainly (Figure 6B, biological process, GO enrichment graph). Most cellular component categories exhibit activity at the cell surface, in the extracellular space, at the membrane, at the membrane of clathrin-coated endocytic vesicles, and in membrane rafts (as indicated by the bar graph and cellular component enrichment graph in Figure 6B). In the case of molecular function categories, the main ones are protein binding and molecular transducing activity associated with cytokine activity, growth factor activity, receptor–ligand binding, and cytokine, chemokine, and chemoattractant binding, as well as CCR chemokine receptor binding (bar graph and graphic molecular function enrichment, Figure 6B). Finally, the biological signaling pathways associated with this set of proteins, according to their enrichment ratio (FDR ≤ 0. 05), were viral protein interaction with cytokine and cytokine receptor, cytokine–cytokine receptor interaction, EGFR tyrosine kinase inhibitor resistance, TNF signaling pathway, MAPK signaling pathway, Ras signaling pathway, PI3K-Akt signaling pathway, JAK-STAT signaling pathway, and chemokine signaling pathway, among others (Figure 6C).

### 3.8. The Mouse Protein Cluster Shows a Relationship Between Chemokine and Cytokine Receptor Binding/Interaction

According to the PPI enrichment analysis of the 19 proteins with significant differences from the murine exosomes (PPI enrichment *p*-value: <1.0 × 10^−16^; 0.700, the highest confidence value), a leading protein cluster related to chemokine receptors, which binds chemokines and interacts with viral proteins and cytokines, was identified (Figure 7A, Table 4, Table 7). Local network (STRING) enrichment analysis confirmed interactions with chemokine ligands, receptors, the JAK-STAT signaling pathway, the interleukin-2 family signaling pathway, and the tumor necrosis factor domain. Cluster analysis showed no interactions between protein subsets and only one big set of proteins (red cluster, Figure 7A). In the GO analysis of biological processes, cell communication, response to stimuli, and biological regulation were identified as the main categories, particularly associated with chemotaxis and migration processes of granulocytes, leukocytes, neutrophils, and lymphocytes, as well as chemokine- and cytokine-mediated signaling pathways (biological process, GO enrichment analysis, Figure 7A). The main cellular components associated with this set of proteins included the extracellular space and the cell surface (outer side of the plasma membrane) (Figure 7B, cellular component, GO enrichment analysis). The associated molecular functions included protein binding, primarily related to cytokine and chemokine activity, as well as cytokine and chemokine receptor binding (CXCR, CCR), growth factor and chemoattractant activity (Figure 7B, molecular function, GO enrichment analysis). Interestingly, the main associated biological signaling pathways were viral protein interaction with cytokines and cytokine receptors, cytokine–cytokine receptor interaction, chemokine, IL-17, and JAK-STAT signaling pathways, according to their enrichment ratios and FDR ≤ 0.05 (Figure 7C, Table 7).

### 3.9. Comparison of Human–Mouse Proteins Showed a Relationship with Cytokine Activity

Finally, for the set of 12 proteins comparable between human and mouse species, PPI enrichment analysis (PPI enrichment *p*-value: <1.11 × 10^−16^; 0, 700 highest confidence value) identified two small clusters of four and two proteins (red and green, Figure 8A, Table 8) primarily associated with lymphocyte chemotaxis. Although there was interaction with a small cluster (two proteins) related to insulin-like growth factor signaling (pathway/binding complex), IL15 also interacted with this cluster. Most pathways and GO terms in the local network cluster (STRING) enrichment analysis showed strong associations with pathways such as chemokine receptor binding, chemokines, insulin-like growth factor-binding protein family 1–6, and the tissue inhibitor of metalloproteinase family, among others (Figure 8A). In the GO analysis, the biological process categories were primarily associated with leukocyte and lymphocyte migration/proliferation, as well as positive regulation of tyrosine phosphorylation of the STAT protein signaling pathway (GO analysis, Figure 8B). The associated cellular components were mainly located in the extracellular space and membrane. They may be associated with the insulin-like growth factor-binding protein complex (Cellular component, GO enrichment analysis, Figure 8B). As previously described, the associated molecular functions will also be primarily related to cytokine activity, receptor–ligand activity, CCR chemokine receptor binding, chemoattractant activity, and growth factor activity (molecular function, GO enrichment analysis, Figure 8B). Finally, the primary biological signaling pathways enriched for this set of proteins, according to FDR, include TNF signaling, viral protein interactions with cytokines and cytokine receptors, cytokine–cytokine receptor interactions, and chemokine signaling pathways. However, other interesting pathways include Toll-like receptor signaling, the JAK-STAT pathway, Rap1 signaling, and Ras signaling (Figure 8C).

## 4. Discussion

In this study, we explored the protein signature of exosomes derived from the blood serum of participants diagnosed with MDD and from mice exposed to the CUMS protocol, a widely used model to mimic depression-like behaviors, with the aim of obtaining translational insights into MDD from an animal model of chronic stress.

MDD is a multifactorial disorder with severe and heterogeneous manifestations across patients, and its high prevalence makes it a major global health concern [80,81]. Although the CUMS model is widely used to study depression-like behaviors in rodents [15,82], the degree of molecular and proteomic homology between clinical depression and depression-like behavior remains unclear. Therefore, as with any in vivo model, its validity is limited to specific behavioral features and their association with selected biochemical and cellular changes at the CNS level [23,83]. In this context, the study of the proteomic content of exosomes in both clinical and murine settings may provide novel insights into the molecular and cellular mechanisms underlying this disorder, their relationship with symptom manifestation and severity, and the development and monitoring of more effective and comprehensive antidepressant treatments aimed at achieving remission and improving patients’ quality of life. Notably, our findings reveal that cytokine and growth factor profiles associated with EVs are significantly altered in both conditions, highlighting EVs as potential mediators and indicators of neuroimmune dysregulation in depression.

Comparison of signaling pathways derived from enrichment analyses revealed that at least eight pathways are shared between humans and mice in MDD and the CUMS model: chemokine signaling, cytokine–cytokine receptor interaction, JAK–STAT signaling, pathways in cancer, Rap1, Ras, and TNF signaling, and viral protein interaction with cytokines and cytokine receptors. Notably, six of these pathways showed an FDR value < 0.05: chemokine signaling, cytokine–cytokine receptor interaction, JAK–STAT, Ras, and TNF signaling, and viral protein interaction with cytokines and their receptors (Table 5, Table 6 and Table 7). These findings suggest that these pathways may be relevant to both MDD and depression-like behavior induced by chronic stress. Importantly, one of the most notable findings of this study is the predominance of downregulated cytokines and growth factors in the EV cargo in both MDD-diagnosed participants and CUMS-exposed mice. This pattern contrasts with the classical view of depression as a purely hyper-inflammatory state [84,85]. Instead, our results support a more nuanced model in which MDD is characterized by dysregulation of immune signaling rather than a uniform increase in inflammatory mediators. The observed reduction in molecules such as BDNF, EGFR, CXCL13, and CX3CL1 suggests a disruption of intercellular communication pathways that are critical for maintaining neuroimmune homeostasis [86,87,88,89,90]. In this context, EVs may reflect systemic alterations and potentially contribute to the propagation of dysfunctional signals between peripheral and central compartments, including mechanisms such as blood–brain barrier (BBB) crossing and neuroimmune communication [91,92,93,94].

The chemokine superfamily (including CXC, CC, C, and CX3C subtypes) has been implicated in processes that may modulate depressive symptomatology, such as immune cell regulation and migration [95,96], blood–brain barrier permeability [97], and synaptic pruning [98]. These chemokines are secreted in response to inflammatory cytokines and selectively recruit monocytes, lymphocytes, and neutrophils by inducing chemotaxis through G protein-coupled receptors (GPCRs) [99]. Chemokines and their receptors are widely expressed in the CNS under both physiological and pathological conditions [100,101,102]. Glial cells—including astrocytes, oligodendrocytes, and microglia—as well as neurons, express several chemokines such as CCL2, CCL3, CCL19, CCL21, CXCL10, and CX3CL1 [103,104,105], which may be upregulated under pathological conditions such as MDD [23]. One of the primary roles of chemokines in the CNS is the regulation of neuronal development and plasticity, influencing key processes such as proliferation, migration, and differentiation of neural progenitor cells. For instance, CX3CL1 is released by mature neurons and astrocytes, while its receptor CX3CR1 is predominantly expressed in microglia [106,107], which plays a central role in synaptic pruning, neurotransmitter modulation, and neurogenesis [108,109]. These processes may be critically involved in neuroplasticity alterations observed in depressive states.

Regarding cytokine–cytokine receptor interactions and TNF signaling pathways, elevated levels of circulating cytokines and their soluble receptors have been consistently reported in MDD patients and animal models [110]. For example, increased plasma levels of IL-6, IL-1β, TNF-α, and C-reactive protein (CRP) have been described. Some cytokines, including IL-6, IL-2, IL-1β, and TNF-α, have been proposed as biomarkers of depressive states or stress, and certain antidepressant treatments have been shown to reduce their levels [111,112,113]. Their effects in depression and stress depend on the specific pathways they regulate; notably, inflammasome activation has been proposed as a key mechanism [114,115]. This pathway responds to elevated levels of damage-associated molecular patterns (DAMPs), triggered by stimuli such as hormonal imbalance and oxidative stress, leading to activation of IL-1β and IL-18, peripheral immune activation, and increased blood–brain barrier permeability. These processes may contribute to astrocytic dysfunction and neuro-inflammation, including microglial activation, which, in turn, promotes the production of chemokines such as CXCL7 and CXCL8. These molecules have been identified in both animal models and human studies and are involved in cascades that modulate neuronal plasticity and neurogenesis [116,117].

Additionally, the interaction between the JAK/STAT and Ras signaling pathways has been associated with depressive disorders and depression-like behaviors in both clinical and preclinical studies [118,119,120]. These pathways often interact with others, such as ERK and MAPK, which were also identified in our enrichment analyses. For example, the Ras/MAPK pathway plays an important role in neuronal plasticity, oxidative stress, cell growth, differentiation, and apoptosis [121,122]. Among these, the JAK/STAT pathway is particularly relevant in neurological disorders due to its direct involvement in inflammatory processes and its distribution across brain regions such as the cortex and hippocampus [123,124,125]. Furthermore, this pathway plays a crucial role in cell growth, survival, development, differentiation, and gene regulation [126]. As a downstream effector of cytokines, growth factors, and reactive oxygen species (ROS), its dysregulation represents a key factor in neurodegenerative and neuropsychiatric conditions [124,125].

Collectively, these findings suggest that protein sets identified in both humans and mice are associated with signaling pathways that may modulate depression and stress [127,128], providing some translational insights and highlighting some similarities between the CUMS model and clinical observations. Although differences in protein expression levels exist, overlapping associations were observed between 36 human and 18 murine proteins involved in these pathways (Figure 5 and Figure 6). Notably, a subset of proteins (Table 7, Figure 8), including CXCL13 (BLC) and CX3CL1 (fractalkine), was consistently downregulated in both species, suggesting conserved mechanisms of neuroimmune communication in response to chronic stress and depressive states. CX3CL1–CX3CR1 signaling plays a critical role in neuron–microglia communication, including microglial activation and synaptic plasticity [129,130]. In contrast, although CXCL13 (BLC) is well known for its role in immune responses and has been associated with complex diseases such as cancer [131,132], its role in psychiatric disorders remains poorly understood. Together, these findings support the hypothesis that alterations in immune signaling and neuro-inflammatory communication are central features of depression [120,127]. Conversely, several proteins displayed species-dependent or even opposite regulation patterns, including ICAM-1, IL-15, MIP-1α, and SCF. These discrepancies highlight important limitations in the direct translation of animal model findings to human conditions. While the CUMS model reproduces key behavioral features and some molecular aspects of depression, our results suggest that systemic immune responses to chronic stress may differ between species, possibly due to differences in physiology, immune system organization, or stress perception.

Another important aspect of this study is the exclusive inclusion of female subjects in both human and animal cohorts. Although this approach prevents the exploration of sex differences, it introduces important biological considerations related to hormonal status. Significant differences were observed in FSH levels and menstrual cycle phase, with a predominance of the follicular phase in the MDD group and the luteal phase in controls. Although the differences were not statistically significant in the latter parameter, they suggest that hormonal fluctuations may influence EV cargo composition [133,134]. Estrogen and progesterone are known to modulate immune responses and hypothalamic–pituitary–adrenal (HPA) axis activity [135,136]. The follicular phase, characterized by lower progesterone levels, has been associated with increased stress sensitivity and altered inflammatory responses [137], whereas the luteal phase may exert stronger immunomodulatory effects [138]. Therefore, the observed EV-associated cytokine profiles may be partially influenced by hormonal status rather than exclusively reflecting disease-related changes. This represents an important factor to consider in data interpretation. Future studies with larger sample sizes and stricter control of menstrual/estrous cycles and hormonal levels will be essential to disentangle endocrine and pathological contributions.

From a methodological perspective, it is important to note that EV isolation was performed using a precipitation-based approach, which enriches exosome-like vesicles but may also co-isolate non-vesicular components, such as protein aggregates and lipoproteins. In addition, the use of antibody-based arrays provides a semi-quantitative assessment of protein expression, which may limit sensitivity and dynamic range compared to quantitative techniques such as ELISA or mass spectrometry. On the other hand, the differences in the results of EV array markers and slot blot for the identification of CD63 may suggest differences in antibody specificity between the array marker and slot blot, as well as in the use of lysed (array marker) or non-lysed (slot blot) exosome-enriched EVs. Therefore, further validation using orthogonal approaches will be necessary to confirm these findings.

Despite these limitations, we consider that our study provides evidence supporting the role of EVs as carriers of biologically relevant information in MDD and chronic stress modeled in mice. The identification of conserved and species-specific EV signatures highlights the complexity of neuroimmune interactions in depression and underscores the importance of integrative approaches combining clinical and experimental models [17,139]. Furthermore, the association of EV cargo with key pathways involved in inflammation [22,134,140], cell communication [24,37], and growth factor signaling [24,25] suggests that EVs may function not only as a source of potential biomarkers but also as active participants in the pathophysiology of depression [23,26,38,39].

## 5. Conclusions

Finally, our findings support the relevance of the protein cargo of serum-derived exosomes from both humans and mice in the context of depression and depression-like behavior, representing an important—although not exclusive—source of information for understanding the molecular mechanisms underlying depressive disorders. Our results suggest that both MDD and chronic stress are associated with a dysregulation of EV-mediated intercellular communication, particularly in pathways related to the immune system and the CNS. This dysregulation is further characterized by a decrease in the expression of cytokines and growth factors, suggesting coordinated alterations in specific signaling networks rather than a simple inflammatory activation. The similarities observed in the bioinformatic analyses indicate partial homology between human and murine expression profiles associated with inflammatory and neuro-inflammatory responses, potentially affecting specific brain regions and cellular substrates within the CNS. This partial overlap between human and murine EV profiles could support the partial translational value of the CUMS model.

Taken together, these findings contribute to a more comprehensive understanding of the molecular mechanisms underlying depression and chronic stress and support the potential of EVs as promising biomarkers in neuropsychiatric disorders.

## 6. Limitations and Perspectives

The present study has several methodological limitations that should be considered when interpreting the results. First, the EV isolation method based on precipitation could be optimized by employing size exclusion chromatography (SEC), which is currently regarded as a more robust approach that improves the purity and yield of small vesicles, such as exosomes, compared to traditional methods. In addition, the quantification of particle number and size should be complemented with orthogonal techniques, such as nanoparticle tracking analysis (NTA) or tunable resistive pulse sensing (TRPS), in order to more accurately validate the EV population under study and to comply with recommended characterization criteria. Regarding molecular and cell line characterization, the analysis of transmembrane markers could be strengthened by incorporating additional techniques, such as Western blotting, including both EV-enriched and EV-depleted (negative) markers, and confirming possible neural and glial origins in human and murine blood serum samples. However, all this must be confirmed by Western blot, mass spectrometry, or flow cytometry, in accordance with the recommendations established by the International Society for Extracellular Vesicles in the MISEV2023 guidelines [69]. This would enable a more comprehensive validation of the identity and purity of the isolated EVs. Another important limitation lies in the scope of the proteomic analysis, which was restricted to the identification of 120 and 96 proteins in human and murine samples, respectively, using antibody arrays targeting cytokines and chemokines. Although this approach enabled the identification of differentially expressed proteins of interest, it does not capture the full proteomic content of EVs. In this context, future studies should incorporate untargeted proteomic strategies, such as liquid chromatography–mass spectrometry (LC–MS), to achieve a more comprehensive characterization of EV cargo in the context of MDD and the CUMS model. These limitations may have influenced the sensitivity and specificity of EV detection and downstream analyses. Despite these limitations, this study represents an initial integrative approach that highlights the potential relevance of EV cargo in the neurobiology of MDD and stress-related responses. Furthermore, it provides a foundation for exploring EVs as a potential source of biomarkers in psychiatric disorders and for advancing our understanding of conserved molecular mechanisms across human and animal models.

## Figures and Tables

**Table 1:** Clinical, biochemical, hormonal and menstrual cycle parameters analyzed: control vs. depressed participants *.

Parameters	CTRL (n = 8)	MDD (n = 12)	t	df	*p*-Value
Clinical					
Age (years)	25.33 ± 5.83	26.33 ± 7.21	0.3733	22	0.7125
Weight (Kg)	55.79 ± 6.83	59.54 ± 13.99	0.8344	22	0.4130
BMI (Kg/m^2^)	22.45 ± 2.57	25.13 ± 5.94	1.429	22	0.1671
**HAMD-17**	**2.41 ± 2.15**	**23.92 ± 4.68**	**14.46**	**22**	**0.0001**
Biochemical					
Glucose (mg/dL)	86.09 ± 4.61	91.17 ± 8.45	1.763	21	0.0924
Total cholesterol (mg/dL)	162.6 ± 29.24	179.8 ± 31.37	1.350	21	0.1915
HDL (mg/dL)	55.55 ± 8.47	50.83 ± 9.50	1.250	21	0.2250
LDL (mg/dL)	90.73 ± 30.92	109.7 ± 26.99	1.569	21	0.1317
Triglyceride acid (mg/dL)	80.36 ± 20.79	96.67 ± 29.85	1.506	21	0.1470
Hormones					
TSH (µLU/mL)	2.48 ± 1.21	3.96 ± 3.61	1.294	21	0.2049
T3 (µg/dL)	116.0 ± 29.70	130.3 ± 14.37	1.495	21	0.1498
T4 (µg/dL)	8.44 ± 1.35	9.03 ± 1.38	1.128	21	0.2719
**FSH (mLU/mL)**	**3.96 ± 1.76**	**5.66 ± 1.78**	**2.286**	**21**	**0.0328**
LH (mLU/mL)	5.41 ± 5.97	7.60 ± 3.53	1.084	21	0.2905
Progesterone (ng/mL)	5.01 ± 8.05	2.83 ± 3.70	0.8452	21	0.4075
Estradiol (pg/mL)	73.90 ± 41.87	54.49 ± 38.39	1.160	21	0.2591
Total testosterone (ng/mL)	0.319 ± 0.147	0.461 ± 0.380	1.164	21	0.2574
Free testosterone (pg/mL)	2.105 ± 2.152	1.470 ± 0.691	0.9715	21	0.3424
Menstrual cycle					
**Parameter**	**CTRL** **(n = 8)**	**MDD** **(n = 12)**	**M-W U**	**IC 95%**	***p*-value**
**Day of the cycle**	**19.43 ± 3.631**	**9.556 ± 2.561**	**11.50**	**−19.00** **–** **−1.000**	**0.0333**
**Parameter**	**n (%)**	**n (%)**	**OR**	**IC 95%**	***p*-value**
Phase of the cycle	Luteal 5 (62.50%)	Luteal 2 (16.67%)	8.333	1.189–51.99	0.0623
	Follicular 3 (37.50%)	Follicular 10 (83.33%)			

* Student’s unpaired two-tailed *t*-test was used to compare CTRL vs. MDD for clinical, biochemical and hormone data; all data are presented as the mean ± standard error of the mean (SEM). The Mann–Whitney U test was used to compare CTRL vs. MDD for the day of the cycle. Fisher’s exact test was used to compare CTRL vs. MDD for the phase of the cycle. Only parameters with significant differences (*p* < 0.05) are shown in bold.

**Table 2:** Hormonal and estrous cycle parameters analyzed: control vs. CUMS protocol *.

Parameters	CTRL (n = 10)	CUMS (n = 10)	t	df	*p*-Value
Hormones					
Corticosterone (pg/mL)	177.1 ± 43.01	251.7 ± 36.05	1.329	22	0.1975
17-β Estradiol (pg/mL)	10.56 ± 3.526	4.184 ± 0.5323	1.788	22	0.0876
Estral cycle					
**Parameter**	**n (%)**	**n (%)**	**OR**	**IC 95%**	***p*-value**
Phase of the cycle	Metaestrus–Diestrus 4 (40%)	Metaestrus–Diestrus 3 (30%)	0.6429	0.1243–3.807	>0.9999
	Proestrus–Estrus 6 (60%)	Proestrus–Estrus 7 (70%)			

* Student’s unpaired two-tailed *t*-test was used to compare CTRL vs. CUMS for hormone data; all data are presented as mean ± standard error of the mean (SEM). Fisher’s exact test was used to compare CTRL vs. CUMS for estrous cycle phase distribution. For comparative purposes, metestrus–diestrus and proestrus–estrus phases are grouped to align murine estrous cycle data with the luteal and follicular phases of the human menstrual cycle, respectively.

**Table 3:** Differentially expressed proteins in human serum-derived exosome-enriched extracellular vesicles *.

Protein	UniProt ID	Gene Symbol	Description	FC	Statistic	*p*-Value	q-Value	Expression Pattern
Acrp30	Q15848	ADIPOQ	Adiponectin	0.7346	U = 22.00	0.045	0.1588	Downregulated
Amphiregulin	P15514	AREG	Amphiregulin	0.5376	U = 17.50	0.012	0.1028	Downregulated
Angiogenin	P03950	ANG	Angiogenin	0.6377	t = 2.713	0.0142	0.1002	Downregulated
Axl	P30530	Axl	Tyrosine-protein kinase receptor UFO	0.4728	U = 21.00	0.023	0.115	Downregulated
**BDNF**	**P23560**	**BDNF**	**Brain-derived neurotrophic factor**	**0.2622**	**t = 5.779**	**0.0000178**	**0.0010**	**Downregulated**
Beta-NGF	P01138	NGF	Beta-nerve growth factor	0.6002	U = 23.00	0.029	0.12	Downregulated
BLC	O43927	CXCL13	C-X-C motif chemokine 13	0.0805	U = 12.50	0.005	0.0666	Strongly downregulated
BMP-4	P12644	BMP4	Bone morphogenetic protein 4	0.1168	U = 15.00	0.008	0.08	Downregulated
**BMP-6**	**P22004**	**BMP6**	**Bone morphogenetic protein 6**	**0.0328**	**U = 8.500**	**<0.001**	**0.03**	**Strongly downregulated**
BTC	P35070	BTC	Probetacellulin	0.4489	U = 22.00	0.024	0.1107	Downregulated
**CK beta 8-1**	**P55773**	**CCL23**	**C-C motif chemokine 23**	**0.0461**	**U = 14.00**	**0.002**	**0.04**	**Strongly downregulated**
CNTF	P26441	CNTF	Ciliary neurotrophic factor	0.2696	U = 17.50	0.012	0.096	Downregulated
EGF	P01133	EGF	Pro-epidermal growth factor	0.3705	U = 24.50	0.025	0.1111	Downregulated
**EGF-R**	**P00533**	**EGFR**	**Epidermal growth factor receptor**	**0.3566**	**t = 4.049**	**0.000753**	**0.0301**	**Downregulated**
ENA-78	P42830	CXCL5	C-X-C motif chemokine 5	0.4892	U = 13.00	0.007	0.084	Downregulated
Eotaxin-1	P51671	CCL11	Eotaxin	0.3718	U = 24.50	0.025	0.1071	Downregulated
Fas/TNFRSF6	P25445	Fas	Tumor necrosis factor receptor superfamily member 6	0.4337	U = 14.00	0.007	0.0763	Downregulated
Flt-3 Ligand	P49771	FLT3L	Fms-related tyrosine kinase 3 ligand	0.2896	U = 19.50	0.023	0.1104	Downregulated
Fractalkine	P78423	CX3CL1	Fractalkine	0.3771	U = 16.00	0.013	0.0975	Downregulated
G-CSF	P09919	CSF3	Granulocyte colony-stimulating factor	2.0938	U = 19.00	0.019	0.1036	Upregulated
GDNF	P39905	GDNF	Glial cell line-derived neurotrophic factor	0.2367	t = 2.595	0.0183	0.1045	Downregulated
GITR ligand	Q9UNG2	TNFSF18	Tumor necrosis factor ligand superfamily member 18	0.7193	U = 22.00	0.038	0.1470	Downregulated
ICAM-1	P05362	ICAM1	Intercellular adhesion molecule 1	0.4759	U = 17.00	0.017	0.102	Downregulated
**IGF-BP-6**	**P24592**	**IGFBP6**	**Insulin-like growth factor-binding protein 6**	**0.3260**	**t = 6.468**	**0.00000439**	**0.0005**	**Downregulated**
IGF-I SR	P08069	IGF1R	Insulin-like growth factor 1 receptor	0.7749	U = 20.00	0.029	0.116	Downregulated
IL-1 R4/ST2	Q01638	IL1RL1	Interleukin-1 receptor-like 1	0.7611	U = 22.00	0.038	0.1425	Downregulated
**MIP-1-alpha**	**P10147**	**CCL3**	**C-C motif chemokine 3**	**4.1522**	**U = 7.000**	**0.002**	**0.0342**	**Strongly upregulated**
MSP-a	P26927	MST1	Hepatocyte growth factor-like protein	0.7047	U = 18.00	0.021	0.1095	Downregulated
PIGF	P49763	PGF	Placenta growth factor	0.7086	U = 16.00	0.011	0.1015	Downregulated
**sgp130**	**P40189**	**II6st**	**Interleukin-6 receptor subunit beta**	**0.3973**	**t = 3.843**	**0.00119**	**0.0285**	**Downregulated**
sTNF RII	P20333	TNFRSF1B	Tumor necrosis factor receptor superfamily member 1B	0.4731	U = 17.00	0.016	0.1010	Downregulated
TIMP-2	P16035	TIMP2	Metalloproteinase inhibitor 2	0.7359	U = 22.00	0.045	0.1542	Downregulated
TPO	P40225	THPO	Thrombopoietin	0.7818	U = 22.00	0.042	0.1527	Downregulated
TRAIL-R3	O14798	TNFRSF1OC	Tumor necrosis factor receptor superfamily member 10	0.6162	U = 12.50	0.004	0.06	Downregulated
TRAIL-R4	Q9UBN6	TNRFSF1OD	Tumor necrosis factor receptor superfamily member 10D	0.7505	U = 17.00	0.015	0.1	Downregulated
uPAR	Q03405	PLAUR	Urokinase plasminogen activator surface receptor	0.7227	U = 23.00	0.049	0.1633	Downregulated

* Student’s unpaired two-tailed *t*-test or Mann–Whitney U test was used to compare chemokine and cytokine expression in the CTRL vs. MDD groups. Proteins were considered significantly different at *p* < 0.05. The q-values were calculated using the Benjamini–Hochberg false discovery rate (FDR) method. Fold change (FC) was calculated as the MDD/CTRL ratio. Expression patterns are classified based on FC, where FC < 1 indicates downregulation and FC > 1 indicates upregulation. Strong regulation is defined as FC < 0.1 or FC > 3. Only proteins with significant differences (q < 0.05) are shown in bold.

**Table 4:** Differentially expressed proteins in mouse serum-derived exosome-enriched extracellular vesicles *.

Protein	UniProt ID	Gene Symbol	Description	FC	Statistic	*p*-Value	q-Value	Expression Pattern
**BLC**	**O55038**	**CXCL13**	**C-X-C motif chemokine 13**	**0.1781**	**t = 5.349**	**0.0017**	**0.0336**	**Downregulated**
**CD30L**	**P32972**	**CD30LG**	**Tumor necrosis factor ligand superfamily member 8**	**0.1003**	**t = 5.848**	**0.0011**	**0.0264**	**Strongly downregulated**
**CGR-2**	**P17515**	**Cxcl10**	**C-X-C motif chemokine 10**	**0.0823**	**t = 5.895**	**0.0010**	**0.0339**	**Strongly downregulated**
CTACK	Q9Z1X0	CCL27	C-C motif chemokine 27	0.3732	t = 3.010	0.0237	0.1896	Downregulated
Eotaxin-1	P48298	CCL11	Eotaxin	0.0004	U = 26.00	0.029	0.1856	Strongly downregulated
Fractalkine	O35188	CX3CL1	Fractalkine	0.1512	t = 3.920	0.0078	0.1069	Downregulated
GM-CSF	P26955	CSF2	Cytokine receptor common subunit beta	0.1994	t = 4.324	0.0049	0.0793	Downregulated
IGFBP-5	Q07079	IGFBP5	Insulin-like growth factor-binding protein 5	0.0821	U = 26.00	0.029	0.174	Strongly downregulated
IL-2	P09535	Igf2	Insulin-like growth factor II	0.2684	t = 3.122	0.0205	0.1968	Downregulated
IL-3 Rb	P26955	Csf2rb	Cytokine receptor common subunit beta	0.2593	t = 3.319	0.016	0.1706	Downregulated
IL-4	P07750	IL4	Interleukin-4	0.1445	U = 0.000	0.029	0.1637	Downregulated
IL-9	P15247	IL9	Interleukin-9	0.2735	t = 3.084	0.0216	0.1885	Downregulated
Leptin	P41160	LEP	Leptin	0.0003	t = 2.972	0.0249	0.1838	Strongly downregulated
Leptin R	Q61215	LEPR	Leptin receptor	0.1917	t = 3.373	0.015	0.18	Downregulated
MIG	P18340	CXCL9	C-X-C motif chemokine 9	0.0020	U = 0.000	0.029	0.1546	Strongly downregulated
Osteopontin	P19008	SPP1	Osteopontin	3.8337	t = -2.883	0.0279	0.1913	Strongly upregulated
**SCF**	**P20826**	**KITLG**	**Ligand for the receptor-type protein tyrosine kinase KIT**	**0.0691**	**t = 12.46**	**0.00001**	**0.0015**	**Strongly downregulated**
**VEGF**	**Q00731**	**VEGFA**	**Vascular endothelial growth factor A, long form**	**0.0233**	**t = 5.927**	**0.00103**	**0.0494**	**Strongly downregulated**

* Student’s unpaired two-tailed *t*-test or Mann–Whitney U test was used to compare chemokine and cytokine expression in the CTRL vs. CUMS groups. Proteins were considered significantly different at *p* < 0.05. The q-values were calculated using the Benjamini–Hochberg false discovery rate (FDR) method. Fold change (FC) was calculated as the CUMS/CTRL ratio. Expression patterns are classified based on FC, where FC < 1 indicates downregulation and FC > 1 indicates upregulation. Strong regulation is defined as FC < 0.1 or FC > 3. Only proteins with significant differences (q < 0.05) are shown in bold.

**Table 5:** Differentially expressed proteins in human–mouse serum-derived exosome-enriched extracellular vesicles *.

Protein	UniProt ID	Gene Symbol	Description	*p*-Value			q-Value			Human FC	Mouse FC	Expression Pattern
				S	G	I	S	G	I			
BLC	O43927	CXCL13	C-X-C motif chemokine 13	0.147	0.033	0.182	0.4246	0.858	1.5773	0.0805	0.1780	Downregulated in both species
Fractalkine	P78423	CX3CL1	Processed fractalkine	0.023	0.099	0.352	0.1495	1.287	1.144	0.3771	0.1512	Downregulated in both species
GM-CSF	P26955	CSF2	Cytokine receptor common subunit beta	0.67	0.041	0.848	0.7573	0.7106	1.0499	0.2360	0.1994	Downregulated in both species
ICAM-1	P05362	ICAM1	Intercellular adhesion molecule 1	0.740	0.945	0.021	0.8187	0.9635	0.546	0.4759	2.0377	Opposite regulation between species
**IGFBP-6**	**P24592**	**IGFBP6**	**Insulin-like growth factor-binding protein 6**	**<0.001**	**<0.001**	**<0.001**	**0.0468**	**0.0468**	**0.0468**	**0.3260**	**0.3567**	**Downregulated in both species**
**IGF-1**	**P05019**	**IGF1**	**Insulin-like growth factor I**	**<0.001**	**0.647**	**0.459**	**0.0234**	**0.9612**	**0.918**	**1.4146**	**0.8711**	**Species-dependent** **Opposite regulation between species**
IL-12 p70	P29459	IL12A	Interleukin-12 subunit alpha	0.019	0.307	0.247	0.1646	0.9977	1.1676	1.1191	0.5457	Opposite regulation between species
IL-15	P40933	IL15	Interleukin-15	0.038	0.847	0.505	0.1976	0.9175	0.9725	0.6965	399.06	Strongly upregulated Opposite regulation between species
MIP-1 α	P10147	CCL3	C-C motif chemokine 3	0.023	0.127	0.044	0.1328	1.1006	0.7626	4.1522	0.3788	Strongly upregulated Opposite regulation between species
**RANTES**	**P13501**	**CCL5**	**C-C motif chemokine 5**	**<0.001**	**0.677**	**0.614**	**0.0156**	**0.9264**	**1.0642**	**0.8692**	**1.9245**	**Species-dependent** **Opposite regulation between species**
SCF	P21583	KITLG	Ligand for the receptor-type protein tyrosine kinase KIT	0.020	0.770	0.337	0.1485	0.8897	1.2517	1.5675	0.0691	Strongly and opposite regulation between species
**TIMP-1**	**P01033**	**TIMP1**	**Metalloproteinase inhibitor 1**	**<0.001**	**0.270**	**0.272**	**0.0117**	**1.2763**	**1.1786**	**0.6927**	**0.9140**	**Species-dependent** **Downregulated in both species**
**TIMP-2**	**P16035**	**TIMP2**	**Metalloproteinase inhibitor 2**	**<0.001**	**0.421**	**0.356**	**0.0093**	**0.9518**	**1.0889**	**0.7359**	**3.6458**	**Species-dependent** **Opposite regulation between species**

* Two-way ANOVA test and Bonferroni’s post hoc test were used to compare chemokine and cytokine expression in the CTRL vs. MDD (human) and CTRL vs. CUMS (mouse) groups. Proteins were considered significantly different at *p* < 0.05. The q-values were calculated using the Benjamini–Hochberg false discovery rate (FDR) method. Fold change (FC) was calculated as the MDD/CTRL and CUMS/CTRL ratio. Expression patterns are classified based on FC, where FC < 1 indicates downregulation and FC > 1 indicates upregulation. Strong regulation was defined as FC < 0.1 or FC > 3. Only proteins with significant differences (q < 0.05) are shown in bold.

**Table 6:** Pathway KEGG enrichment analysis of the human protein set *.

Gene Set	Description	Size	Expect	Ratio	*p*-Value	FDR
hsa04060	Cytokine–cytokine receptor interaction	275	1.0601	16.036	1.02 × 10^−17^	3.57 × 10^−15^
hsa04061	Viral protein interaction with cytokine and cytokine receptor	90	0.34695	25.94	2.89 × 10^−11^	5.07 × 10^−9^
hsa04010	MAPK signaling pathway	294	1.1334	8.8231	7.81 × 10^−8^	9.1389 × 10^−6^
hsa04151	PI3K-Akt signaling pathway	344	1.3261	7.5407	3.41 × 10^−7^	0.000029888
hsa04014	Ras signaling pathway	227	0.8751	7.9991	0.000018806	0.0013202
hsa04668	TNF signaling pathway	110	0.42406	11.791	0.000055379	0.0032397
hsa01521	EGFR tyrosine kinase inhibitor resistance	78	0.30069	13.303	0.00021	0.01053
hsa04012	ErbB signaling pathway	83	0.31997	12.501	0.00026694	0.011712
hsa04630	JAK-STAT signaling pathway	158	0.6091	8.2089	0.00030607	0.011937
hsa04062	Chemokine signaling pathway	178	0.6862	7.2865	0.00053012	0.017728
hsa05200	Pathways in cancer	515	1.9854	4.0295	0.00055556	0.017728
hsa04015	Rap1 signaling pathway	205	0.79029	6.3268	0.0010062	0.029432
hsa05218	Melanoma	70	0.26985	11.117	0.002378	0.064207
hsa05214	Glioma	75	0.28913	10.376	0.0028957	0.072599
hsa05210	Colorectal cancer	85	0.32768	9.1553	0.0041272	0.096577
hsa04657	IL-17 signaling pathway	90	0.34695	8.6467	0.0048459	0.10631
hsa04640	Hematopoietic cell lineage	92	0.35466	8.4587	0.0051532	0.1064
hsa05215	Prostate cancer	95	0.36623	8.1916	0.005636	0.1099
hsa04510	Focal adhesion	193	0.74402	5.3762	0.0060657	0.11206
hsa05205	Proteoglycans in cancer	198	0.7633	5.2404	0.0066353	0.11645

* Over-representation analysis (ORA) was performed using the WebGestalt platform. Enrichment analysis included a minimum of 5 IDs and a maximum of 2000 IDs. Multiple testing correction was applied using the Benjamini–Hochberg false discovery rate (FDR), and pathways with an adjusted *p*-value < 0.05 were considered statistically significant.

**Table 7:** Pathway KEGG enrichment analysis of the mouse protein set *.

Gene Set	Description	Size	Expect	Ratio	*p*-Value	FDR
mmu04060	Cytokine–cytokine receptor interaction	294	0.515	25.243	1.57 × 10^−17^	5.49 × 10^−15^
mmu04061	Viral protein interaction with cytokine and cytokine receptor	95	0.16641	42.064	1.12 × 10^−10^	1.95 × 10^−8^
mmu04062	Chemokine signaling pathway	192	0.33633	20.813	1.58 × 10^−8^	1.8436 × 10^−6^
mmu05310	Asthma	25	0.043792	68.505	9.9087 × 10^−6^	0.00086454
mmu04630	JAK-STAT signaling pathway	171	0.29954	13.354	0.00018103	0.012636
mmu04657	IL-17 signaling pathway	93	0.16291	18.415	0.00051984	0.030238
mmu05200	Pathways in cancer	542	0.94942	5.2664	0.0018241	0.090947
mmu04151	PI3K-Akt signaling pathway	364	0.63762	6.2734	0.0030839	0.13454
mmu04014	Ras signaling pathway	235	0.41165	7.2878	0.007348	0.28494
mmu04640	Hematopoietic cell lineage	94	0.16466	12.146	0.011449	0.39955
mmu04620	Toll-like receptor signaling pathway	104	0.18218	10.978	0.013886	0.41958
mmu04010	MAPK signaling pathway	301	0.52726	5.6898	0.014427	0.41958
mmu04668	TNF signaling pathway	115	0.20145	9.9283	0.016806	0.45117
mmu05205	Proteoglycans in cancer	202	0.35384	5.6522	0.047652	1
mmu04015	Rap1 signaling pathway	214	0.37486	5.3353	0.052851	1
mmu05219	Bladder cancer	41	0.07182	13.924	0.069507	1
mmu05163	Human cytomegalovirus infection	253	0.44318	4.5128	0.071068	1
mmu04672	Intestinal immune network for IgA production	43	0.075323	13.276	0.072779	1
mmu04370	VEGF signaling pathway	58	0.1016	9.8427	0.096976	1
mmu05330	Allograft rejection	60	0.1051	9.5146	0.10016	1

* Over-representation analysis (ORA) was performed using the WebGestalt platform. Enrichment analysis included a minimum of 5 IDs and a maximum of 2000 IDs. Multiple testing correction was applied using the Benjamini–Hochberg false discovery rate (FDR), and pathways with an adjusted *p*-value < 0.05 were considered statistically significant.

**Table 8:** Pathway KEGG enrichment analysis of the human–mouse protein set *.

Gene Set	Description	Size	Expect	Ratio	*p*-Value	FDR
hsa04668	TNF signaling pathway	110	0.12722	31.442	0.00000451	0.0010461
hsa04060	Cytokine–cytokine receptor interaction	275	0.31804	15.721	5.9609 × 10^−6^	0.0010461
hsa05323	Rheumatoid arthritis	86	0.09946	30.163	0.00010434	0.010486
hsa04061	Viral protein interaction with cytokine and cytokine receptor	90	0.10409	28.822	0.0001195	0.010486
hsa05143	African trypanosomiasis	34	0.039322	50.863	0.00065438	0.039685
hsa05164	Influenza A	162	0.18736	16.012	0.00067838	0.039685
hsa04062	Chemokine signaling pathway	178	0.20586	14.573	0.00089301	0.044778
hsa05144	Malaria	46	0.0532	37.594	0.0011986	0.051015
hsa05417	Lipid and atherosclerosis	203	0.23477	12.778	0.0013081	0.051015
hsa05200	Pathways in cancer	515	0.59561	6.7159	0.0018273	0.064137
hsa05142	Chagas disease	97	0.11218	17.828	0.0052293	0.16232
hsa04620	Toll-like receptor signaling pathway	100	0.11565	17.293	0.0055494	0.16232
hsa04066	HIF-1 signaling pathway	108	0.1249	16.012	0.0064466	0.17406
hsa04630	JAK-STAT signaling pathway	158	0.18273	10.945	0.013428	0.33666
hsa04015	Rap1 signaling pathway	205	0.23709	8.4358	0.022007	0.49257
hsa05166	Human T-cell leukemia virus 1 infection	213	0.24634	8.1189	0.023648	0.49257
hsa05163	Human cytomegalovirus infection	214	0.24749	8.081	0.023857	0.49257
hsa04014	Ras signaling pathway	227	0.26253	7.6182	0.026641	0.5195
hsa04960	Aldosterone-regulated sodium reabsorption	36	0.041635	24.019	0.040893	0.71767
hsa05330	Allograft rejection	36	0.041635	24.019	0.040893	0.71767

* Over-representation analysis (ORA) was performed using the WebGestalt platform. Enrichment analysis included a minimum of 5 IDs and a maximum of 2000 IDs. Multiple testing correction was applied using the Benjamini–Hochberg false discovery rate (FDR), and pathways with an adjusted *p*-value < 0.05 were considered statistically significant.

## Data Availability

The original contributions presented in this study are included in this article/Supplementary Materials. Further inquiries can be directed to the corresponding author. However, the participants did not agree to publish their complete information; they only agreed to publish the study results without data that could identify them.

## Supplementary Materials

The following supporting information can be downloaded at: https://www.mdpi.com/article/10.3390/cells15121042/s1.

## Abbreviations

The following abbreviations are used in this manuscript:

MDD	Major depressive disorder
CNS	Central nervous system
CUMS	Chronic unpredictable mild stress
EVs	Extracellular vesicles
CTRL	Healthy control group
PPI	Protein–protein interactions analysis
GO	Gene ontology analysis
DSM-5	*Diagnostic and Statistical Manual of Mental Disorders*
nm	Nanometers
BBB	Blood–brain barrier
CSF	Cerebrospinal fluid
HDRS	Hamilton Depression Rating Scale
g	Grams
cm	Centimeters
h	Hours
rpm	Revolutions per minute
am	Ante meridiem
mL	Milliliter
μm	Micrometers
min	Minutes
xg	g-force, relative centrifugal force
PBS	Phosphate-buffered saline solution
RIPA	Radio-Immunoprecipitation Assay Buffer, lysis and extraction buffer
HRP	Horseradish peroxidase solution
kV	Kilovolts
FSH	Follicle-stimulating hormone
HDL	High-density lipoprotein
LDL	Low-density lipoprotein
TSH	Thyroid-stimulating hormone
T3	Triiodothyronine
T4	Thyroxine
LH	Luteinizing hormone
ANOVA	Analysis of variance
