# Supplementary material for: Signatures in the Protein Content of Human and Murine Blood Serum Exosomes, in the Context of Major Depressive Disorder, Are Associated with Cytokine Activity
Source: Cells. 2026 Jun 6;15(12):1042. doi: 10.3390/cells15121042 (PMC13297292; doi:10.3390/cells15121042)
Supplement: Supplementary file 1 [file cells-15-01042-s001.zip › cells-4149353-supplementary figures.pdf]

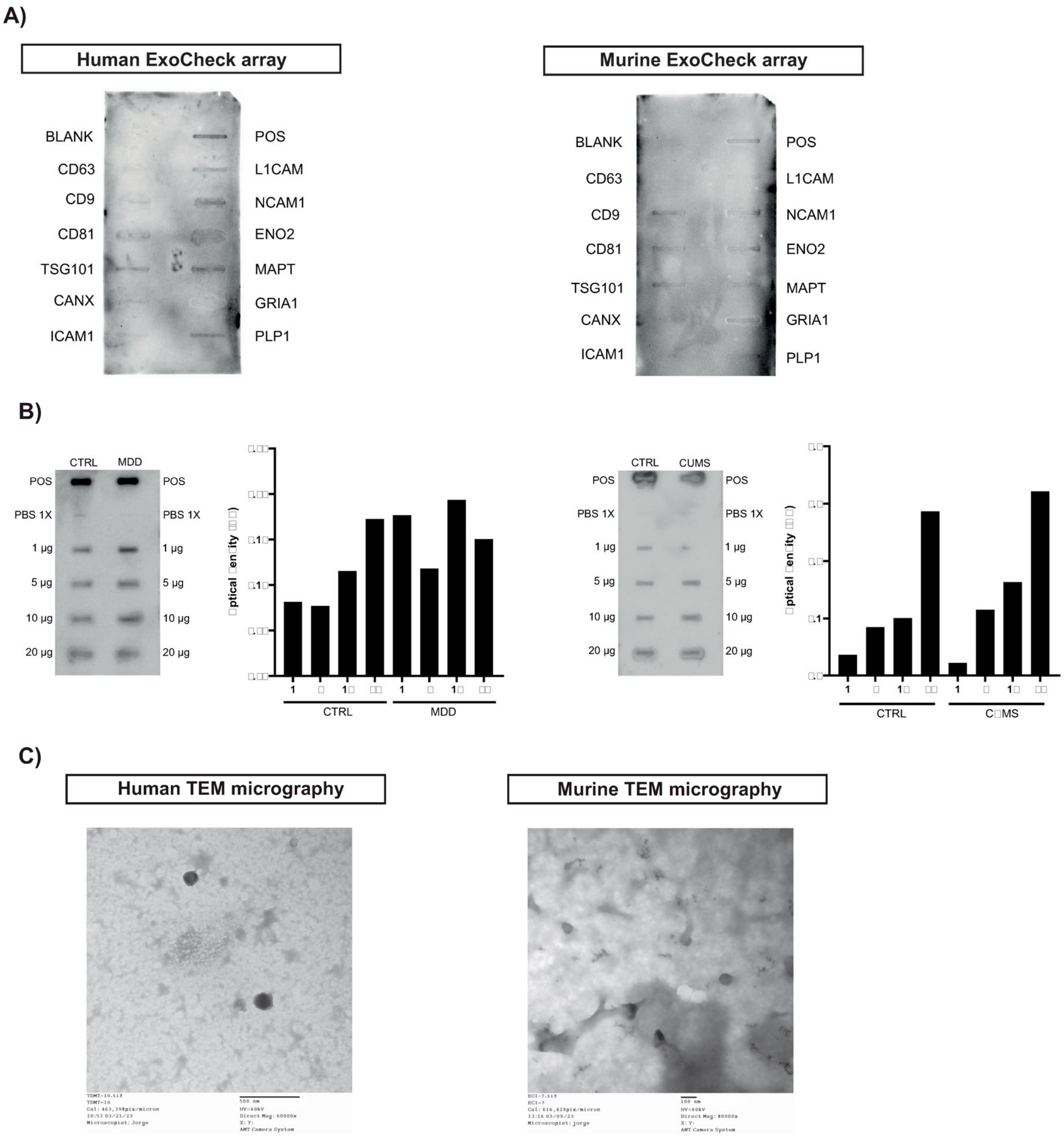

Supplementary Figure 1. Human and murine exosome caracterization: Exo-Check antibody array (Neuro) standard kit and Transmission electron microscopy (TEM). **A)** Presence of exosomal markers in human and murine exosomes samples, and optical density analysis. **B)** CD63 Slot blot and optical density analysis. **C)** Micrography TEM analysis revealed the presence of small EV (exosomes), ranging from 30 to 180 nm in both human and murine exosomes samples.

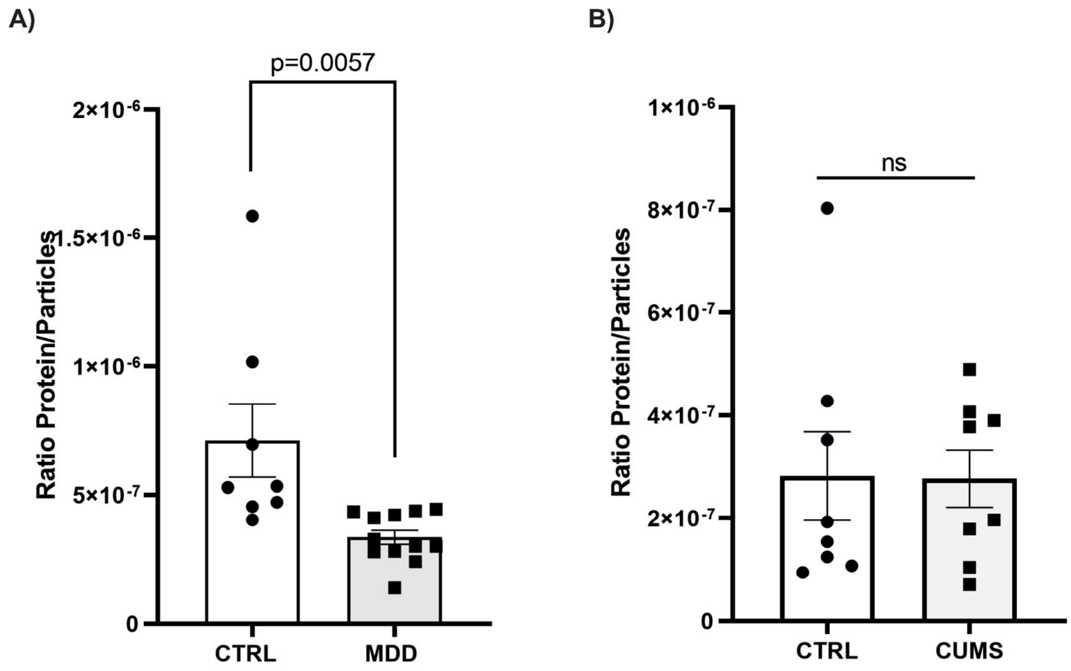

Supplementary Figure 2. Protein/Particle ratio: CTRL vs MDD and CTRL vs CUMS. A) Protein/Particle Ratio in human samples, a significant decrease of 52.94% in the ratio was identified for the MDD group (p = 0.0057). **B)** Protein/Particle Ratio in murine samples, no significant differences was identified between CTRL and CUMS groups.

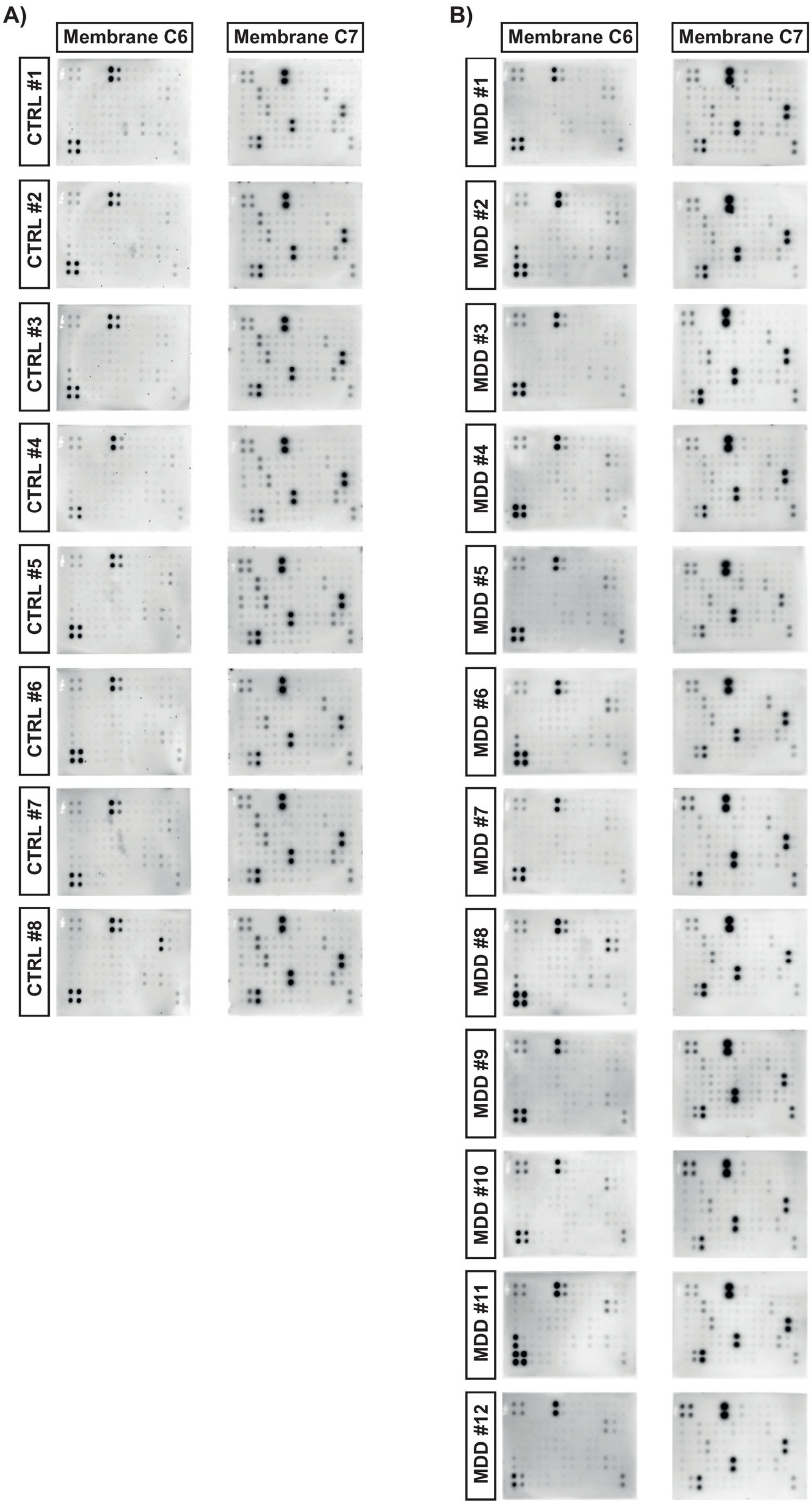

Supplementary Figure 3. Semi-quantitative analysis of human exosome content: Human cytokine array C-1000 membranes. **A)** C6 and C7 membranes lysed exosomes CTRL group. **B)** C6 and C7 membranes lysed exosomes MDD group.

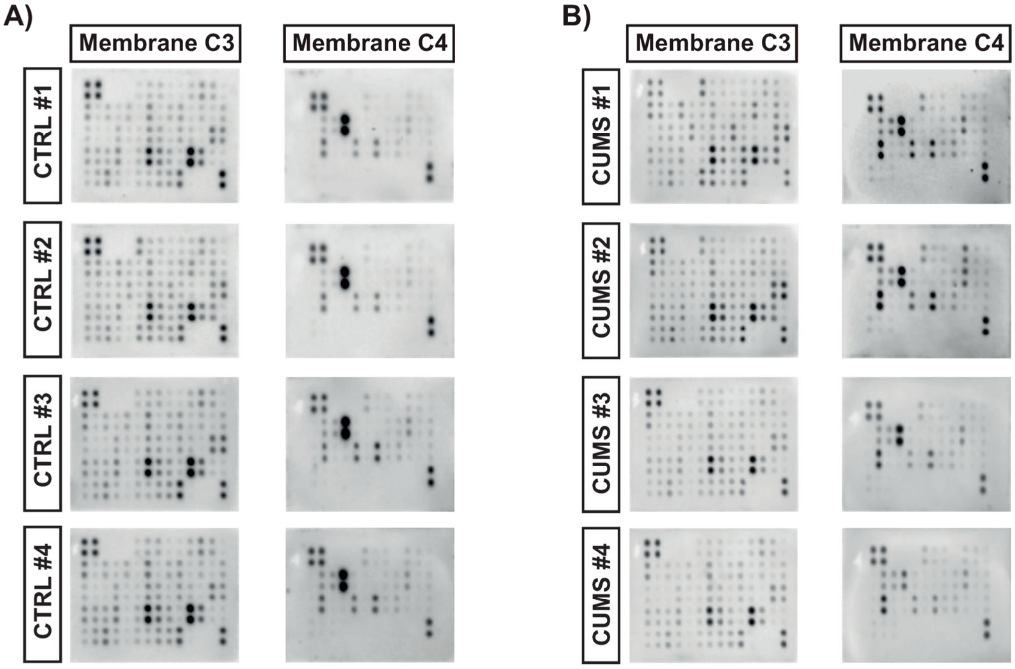

Supplementary Figure 4. Semi-quantitative analysis of murine exosome content: Mouse cytokine array C-1000 membranes. **A)** C3 and C4 membranes lysed exosomes CTRL group. **B)** C3 and C4 membranes lysed exosomes CUMS group.
