# Supplementary material for: Signatures in the Protein Content of Human and Murine Blood Serum Exosomes, in the Context of Major Depressive Disorder, Are Associated with Cytokine Activity
Source: Cells. 2026 Jun 6;15(12):1042. doi: 10.3390/cells15121042 (PMC13297292; doi:10.3390/cells15121042)
Supplement: Supplementary file 1 [file cells-15-01042-s001.zip › Supplementary information_Cells_MDPI_4149353.pdf]

Supplementary information.

2.8.2. Bioinformatic and proteomic data analysis.

Software/Database: UniProt database (Universal Protein Resource)
Version: Release 2024_01
Website: https://www.uniprot.org/
Date: 24/11/2024

Software/Database: STRING database (Functional Protein Association Networks)
Version: STRING 11.5
Website: https://string-db.org/
Date: 24/11/2024

Software/Database: WEBGESTALT database (WEB-based Gene SeT Analysis Toolkit)
Version: 2024
Website: http://www.webgestalt.org/
Date: 24/11/2024

Software/Database: BioRender
Version: 2025
Website: https://app.biorender.com/
Date: 08/12/2025

Software/Database: SigmaPlot
Version: SigmaPlot 15.0
Date: 10/10/2024

Software/Database: GraphPad Prims
Version: Prism 8.0.2
Date: 10/10/2024

Software/Database: Adobe Illustrator CC 2019
Version: 23.0.2 (64-bit)
Date: 08/12/2025

Software/Database: Connected Papers
Version: Free
Website: https://www.connectedpapers.com/
Date: June/2024 – April/2026

Figure 1. A), B) and C) panel, use BioRender and Adobe Illustrator.

Figure 2. A) panel, use Adobe Illustrator; B) panel, GraphPad Prism and SigmaPlot.

Figure 3. A), B), E), and F) panel, use GraphPad Prism and SigmaPlot; C), D), G), and H) panel, use Adobe Illustrator.

Figure 4. A) panel, use BioRender and Adobe Illustrator; C) and D) panel, use GraphPad Prism and SigmaPlot.

Figure 5. A) and B) panel, use STRING database and WEBGESTALT database; C) panel use WEBGESTALT database.

Figure 6. A) and B) panel, use STRING database and WEBGESTALT database; C) panel use WEBGESTALT database.

Figure 7. A) and B) panel, use STRING database and WEBGESTALT database; C) panel use WEBGESTALT database.

Figure 8. Use BioRender and Adobe Illustrator.
