# Supplementary material for: Signatures in the Protein Content of Human and Murine Blood Serum Exosomes, in the Context of Major Depressive Disorder, Are Associated with Cytokine Activity
Source: Cells. 2026 Jun 6;15(12):1042. doi: 10.3390/cells15121042 (PMC13297292; doi:10.3390/cells15121042)
Supplement: Supplementary file 1 [file cells-15-01042-s001.zip › Supplementary Table S1_Exo-MDD_Cells.pdf]

Supplementary Table S1. Statistical results of 120 chemokines/cytokines from human exosomes.

Protein	Description	Statistic	p-Value	q-Value	Fold Change
Acrp30	Adiponectin	U = 22.00	0.045	0.158823529	0.734652133
AgRP	Agouti-related protein	t = -0.348	0.732	0.813333333	1.090492773
Amphiregulin	Amphiregulin	U = 17.50	0.012	0.102857143	0.537622779
Angiogenin	Angiogenin	t = 2.713	0.0142	0.100235294	0.637734092
Angiopoietin-2	Angiopoietin-2	U = 28.00	0.121	0.259285714	0.779404259
Axl	Tyrosine-protein kinase receptor UFO	U = 21.00	0.023	0.115	0.472804571
BDNF	Neurotrophic factor BDNF precursor form	t = 5.779	0.0000178	0.001068	0.262225255
Beta-NGF	Beta-nerve growth factor	U = 23.00	0.029	0.12	0.600265655
bFGF	Fibroblast growth factor 2	U = 28.00	0.091	0.2184	0.829418681
BLC	C-X-C motif chemokine 13	U =12.50	0.005	0.066666667	0.080596598
BMP-4	Bone morphogenetic protein 4	U = 15.00	0.008	0.08	0.116816634
BMP-6	Bone morphogenetic protein 6	U = 8.500	0.001	0.03	0.032885672
BTC	Probetacellulin	U = 22.00	0.024	0.110769231	0.448919852
CCL28	C-C motif chemokine 28	U = 47.50	0.967	0.967	1.611557047
CK beta 8-1	C-C motif chemokine 23	U = 14.00	0.002	0.04	0.046135068
CNTF	Ciliary neurotrophic factor	U = 17.50	0.012	0.096	0.26962527
CTACK	C-C motif chemokine 27	U = 46.00	0.877	0.915130435	1.067523395
Dtk	Tyrosine-protein kinase receptor TYRO3	U = 26.00	0.09	0.220408163	1.165331142
EGF	Pro-epidermal growth factor	U = 24.50	0.025	0.111111111	0.370569513
EGF-R	Epidermal growth factor receptor	t = 4.049	0.000753	0.03012	0.356667136
ENA-78	C-X-C motif chemokine 5	U = 13.00	0.007	0.084	0.489205563
Eotaxin	Eotaxin	U = 24.50	0.025	0.107142857	0.371802811
Eotaxin-2	C-C motif chemokine 24	t = 0.766	0.454	0.5675	0.766411187
Eotaxin-3	C-C motif chemokine 26	t = 0.825	0.42	0.553846154	0.762509119
Fas/TNFRSF6	Tumor necrosis factor receptor superfamily member 6	U = 14.00	0.007	0.076363636	0.433796832
FGF-4	Fibroblast growth factor 4	U = 25.00	0.075	0.2	0.7199307
FGF-6	Fibroblast growth factor 6	t = 1.319	0.204	0.349714286	0.641837853
FGF-7	Fibroblast growth factor 7	t = 1.654	0.115	0.250909091	0.403812452
FGF-9	Fibroblast growth factor 9	U = 30.00	0.162	0.308571429	0.805764162
Flt-3 Ligand	Fms-related tyrosine kinase 3 ligand	U = 19.50	0.023	0.1104	0.289668688
Fractalkine	Fractalkine	U = 16.00	0.013	0.0975	0.377181204
GCP-2	C-X-C motif chemokine 6	t = 1.825	0.0847	0.220956522	0.371842951
G-CSF	Granulocyte colony-stimulating factor	U = 19.00	0.019	0.103636364	2.093828881
GDNF	Glial cell line-derived neurotrophic factor	t = 2.595	0.0183	0.104571429	0.236726077
GITR	Tumor necrosis factor receptor superfamily member 18	U = 25.00	0.07	0.204878049	0.889225638
GITR ligand	Tumor necrosis factor ligand superfamily member 18	U = 22.00	0.038	0.147096774	0.719315056
GM-CSF	Granulocyte-macrophage colony-stimulating factor	U = 33.00	0.174	0.321230769	0.236006315

GRO	Growth-regulated alpha protein	t = 0.887	0.387	0.521797753	0.745005204
GRO-alpha	Growth-regulated alpha protein	U = 33.00	0.225	0.369863014	0.931559165
HCC-4	C-C motif chemokine 16	U = 32.00	0.196	0.340869565	0.699787182
HGF	Hepatocyte growth factor	U = 31.00	0.169	0.316875	0.709627926
I-309	C-C motif chemokine 1	U = 35.00	0.258	0.407368421	0.232584012
ICAM-1	Intercellular adhesion molecule 1	U = 17.00	0.017	0.102	0.47590143
ICAM-3	Intercellular adhesion molecule 3	U = 38.00	0.44	0.561702128	1.227786142
IFN-gamma	Interferon gamma	U = 31.50	0.151	0.302	0.45245175
IGFBP-1	Insulin-like growth factor-binding protein 1	U = 32.00	0.217	0.361666667	1.14960517
IGFBP-2	Insulin-like growth factor-binding protein 2	U = 28.00	0.122	0.256842105	0.615804121
IGF-BP-3	Insulin-like growth factor-binding protein 3	U = 23.00	0.054	0.170526316	0.786003998
IGFBP-4	Insulin-like growth factor-binding protein 4	U = 31.50	0.151	0.29704918	0.506075237
IGF-BP-6	Insulin-like growth factor-binding protein 6	t = 6.468	0.00000439	0.0005268	0.326044286
IGF-I	Insulin-like growth factor 1	U = 44.00	0.757	0.825818182	1.414670404
IGF-I SR	Insulin-like growth factor 1 receptor	U = 20.00	0.029	0.116	0.774921
IL-1 R4/ST2	Interleukin-1 receptor-like 1	U = 22.00	0.038	0.1425	0.761199055
IL-1 RI	Interleukin-1 receptor-like 1	U = 27.00	0.094	0.221176471	0.747058782
IL-10	Interleukin-10	t = -0.738	0.47	0.575510204	1.322134397
IL11	Interleukin-11	U = 24.00	0.052	0.168648649	1.439717127
IL12-p40	Interleukin-12 subunit beta	U = 27.00	0.094	0.216923077	0.953391332
IL12-p70	Interleukin-12 subunit alpha	U = 25.00	0.067	0.201	1.119145872
IL-13	Interleukin-13	U = 44.50	0.784	0.847567568	0.881118713
IL-15	Interleukin-15	U = 39.50	0.506	0.6072	0.696510894
IL-16	Pro-interleukin-16	U = 31.00	0.175	0.313432836	0.644525456
IL17	Interleukin-17A	U =28.00	0.07	0.2	1.593652073
IL-1alpha	Interleukin-1 alpha	t = 1.038	0.313	0.475443038	0.65563216
IL-1beta	Interleukin-1 beta	t = 1.899	0.0738	0.205953488	0.4725268
IL-1ra	Interleukin-1 receptor antagonist protein	U = 38.00	0.41	0.546666667	0.544089568
IL-2	Interleukin-2	U = 44.00	0.742	0.816880734	0.688541358
IL-2 Ra	Interleukin-2 receptor subunit alpha	U = 34.00	0.278	0.427692308	0.802995087
IL-3	Interleukin-3	t = 1.761	0.0952	0.21554717	0.440309973
IL-4	Interleukin-4	U = 42.00	0.627	0.730485437	0.707128595
IL-5	Interleukin-5	U = 46.50	0.905	0.936206897	1.262087374
IL-6	Interleukin-6	U = 31.50	0.174	0.316363636	4.494435304
IL-6 R	Interleukin-6 receptor subunit alpha	t = 1.976	0.0637	0.196	0.691505267
IL-7	Interleukin-7	U = 47.00	0.928	0.943728814	1.337345583
IL8	Interleukin-8	U = 38.00	0.439	0.572608696	1.095667324
I-TAC	C-X-C motif chemokine 11	t = -1.511	0.148	0.301016949	1.827217998
Leptin	Leptin	U = 43.00	0.7	0.79245283	2.678035261
LIGHT	Tumor necrosis factor ligand superfamily member 14	U = 35.00	0.315	0.4725	1.655879916

Lymphotactin	Lymphotactin	U = 43.00	0.699	0.798857143	1.411890015
MCP-1	C-C motif chemokine 2	U = 36.50	0.369	0.527142857	1.017823189
MCP-2	C-C motif chemokine 8	U = 39.00	0.473	0.573333333	0.929783199
MCP-3	C-C motif chemokine 7	U = 45.00	0.808	0.865714286	0.907078413
MCP-4	C-C motif chemokine 13	U = 37.00	0.386	0.526363636	0.872355521
M-CSF	Macrophage colony-stimulating factor 1	U = 46.50	0.907	0.93025641	1.245623702
MDC	C-C motif chemokine 22	U = 40.50	0.536	0.636831683	0.843379298
MIF	Macrophage migration inhibitory factor	U =35.00	0.315	0.466666667	1.229075064
MIG	C-X-C motif chemokine 9	U = 34.50	0.266	0.414545455	0.56108329
MIP-1-alpha	C-C motif chemokine 3	U =7.00	0.002	0.034285714	4.152235402
MIP-1-beta	C-C motif chemokine 4	U = 25.00	0.074	0.201818182	0.782852812
MIP-1-delta	C-C motif chemokine 15	t = 1.316	0.205	0.346478873	0.609716108
MIP-3-alpha	C-C motif chemokine 20	U = 46.00	0.875	0.921052632	0.955096407
MIP-3-beta	C-C motif chemokine 19	U = 38.00	0.439	0.566451613	1.051171168
MSP-a	Hepatocyte growth factor-like protein	U = 18.00	0.021	0.109565217	0.704722646
NAP-2	Platelet basic protein	t = 0.785	0.442	0.558315789	0.782045528
NT-3	Neurotrophin-3	t = -0.977	0.342	0.494457831	1.631913266
NT-4	Neurotrophin-4	U = 31.00	0.188	0.331764706	0.850685732
Oncostatin M	Oncostatin-M	U = 42.00	0.641	0.739615385	1.066681756
Osteoprotegerin	Tumor necrosis factor receptor superfamily member 11B	U = 30.00	0.159	0.307741935	0.776696739
PARC	C-C motif chemokine 18	U = 37.00	0.381	0.531627907	2.690814568
PDGF-BB	Platelet-derived growth factor subunit B	t = 0.0878	0.931	0.938823529	0.981304219
PLGF	Placenta growth factor	U = 16.00	0.011	0.101538462	0.708663645
RANTES	C-C motif chemokine 5	t = 0.746	0.465	0.575257732	0.869287442
SCF	Kit ligand	t = -1.019	0.322	0.471219512	1.567580315
SDF-1	Stromal cell-derived factor 1	U = 28.00	0.106	0.235555556	0.662152223
sgp130	Interleukin-6 receptor subunit beta	t = 3.843	0.00119	0.02856	0.397300866
sTNF RII	Tumor necrosis factor receptor superfamily member 1B	U = 17.00	0.016	0.101052632	0.47311768
sTNF-RI	Tumor necrosis factor receptor superfamily member 1A	U = 36.50	0.369	0.520941176	1.081451989
TARC	C-C motif chemokine 17	U = 26.50	0.087	0.22212766	0.698291734
TECK	C-C motif chemokine 25	t = -0.372	0.714	0.800747664	1.160780978
TGF-beta 1	Transforming growth factor beta-1 proprotein	U = 38.00	0.384	0.529655172	0.950830762
TGF-beta 3	Transforming growth factor beta-3 proprotein	U =33.00	0.225	0.364864865	0.901486052
TIMP-1	Metalloproteinase inhibitor 1	t = 1.799	0.0889	0.22225	0.692796497
TIMP-2	Metalloproteinase inhibitor 2	U =22.00	0.045	0.154285714	0.735991966
TNF-alpha	Tumor necrosis factor	U =42.50	0.601	0.707058824	0.935331799
TNF-beta	Lymphotoxin-alpha	U =46.00	0.856	0.909026549	0.978070801
TPO	Thrombopoietin	U =22.00	0.042	0.152727273	0.781870845
TRAIL-R3	Tumor necrosis factor receptor superfamily member 10C	U = 12.00	0.004	0.06	0.616258739
TRAIL-R4	Tumor necrosis factor receptor superfamily member 10D	U = 17.00	0.015	0.1	0.750556851

uPAR	Urokinase plasminogen activator surface receptor	U = 23.00	0.049	0.163333333	0.722749713
VEGF	Vascular endothelial growth factor A, long form	U = 33.00	0.225	0.36	2.424650606
VEGF-D	Vascular endothelial growth factor D	U = 29.00	0.13	0.268965517	1.2511851

* Student's unpaired two-tailed t-test or Mann-Whitney U test were used to compare chemokine and cytokine expression in CTRL vs MDD group. Proteins were considered significantly different at $p < 0.05$. The q-values were calculated using the Benjamini–Hochberg false discovery rate (FDR) method. Fold Change (FC) were calculated as MDD/CTRL ratio.
