# Supplementary material for: Signatures in the Protein Content of Human and Murine Blood Serum Exosomes, in the Context of Major Depressive Disorder, Are Associated with Cytokine Activity
Source: Cells. 2026 Jun 6;15(12):1042. doi: 10.3390/cells15121042 (PMC13297292; doi:10.3390/cells15121042)
Supplement: Supplementary file 1 [file cells-15-01042-s001.zip › Supplementary Table S2_Exo-MDD_Cells.pdf]

Supplementary Table S2. Statistical results of 96 chemokines/cytokines from mouse exosomes.

Protein	Description	Statistic	*p*-Value	q-Value	Fold Change
Axl	Tyrosine-protein kinase receptor UFO	U = 5.000	0.486	0.598153846	2.272823601
bFGF	Fibroblast growth factor 2	t = -1.504	0.183	0.462315789	4.078361774
BLC	C-X-C motif chemokine 13	t = 5.349	0.00175	0.0336	0.178120119
CD30/TNFRSF8	Tumor necrosis factor ligand superfamily member 8	t = 2.356	0.0566	0.27168	0.00134325
CD30L	Tumor necrosis factor receptor superfamily member 8	t =5.848	0.0011	0.0264	0.10037557
CD40	Tumor necrosis factor receptor superfamily member 5	U = 2.000	0.114	0.420923077	0.000947924
CRG-2	C-X-C motif chemokine 10	t = 5.895	0.00106	0.03392	0.082348706
CTACK	C-C motif chemokine 27	t = 3.010	0.0237	0.1896	0.37321763
CXCL16	C-X-C motif chemokine 16	t = 2.349	0.0572	0.261485714	0.41848523
DPPIV/CD26	Dipeptidyl peptidase 4	U = 4.000	0.343	0.567724138	3488.76775
Dtk	Tyrosine-protein kinase receptor TYRO3	U = 5.000	0.486	0.590582278	64.84678658
Eotaxin	Eotaxin	U = 0.000	0.029	0.1856	0.00049137
Eotaxin-2	C-C motif chemokine 24	t = -0.0765	0.941	0.971354839	1.026758345
E-Selectin	E-selectin	U = 6.000	0.686	0.765767442	2161.80325
FAS ligand	Tumor necrosis factor ligand superfamily member 6	U = 4.000	0.343	0.558101695	1.032528307
Fcg RIIB	Low affinity immunoglobulin gamma Fc region receptor II	U = 8.000	1	1.021276596	7.107490236
Flt-3 Ligan	Fms-related tyrosine kinase 3 ligand	t = -1.504	0.183	0.450461538	6.698718741
Fractalkine	Fractalkine	t = 3.920	0.0078	0.106971429	0.15129565
G-CSF	Granulocyte colony-stimulating factor	U = 4.000	0.343	0.5488	1.874162088
GITR	Tumor necrosis factor receptor superfamily member 18	t = -1.599	0.161	0.454588235	54.33808468
GM-CSF	Granulocyte-macrophage colony-stimulating factor	t = 4.324	0.00496	0.07936	0.199433144
HGF R	Hepatocyte growth factor	U = 7.000	0.886	0.955685393	2.156475278
ICAM-1	Intercellular adhesion molecule 1	t = -1.833	0.116	0.412444444	2.037703704
IFN-gamma	Interferon gamma	U = 5.500	0.486	0.5832	1.790255403
IGFBP-2	Insulin-like growth factor-binding protein 2	t = -1.283	0.247	0.483918367	1.783053858
IGF-BP-3	Insulin-like growth factor-binding protein 3	t = 1.446	0.198	0.452571429	0.590538219
IGF-BP-5	Insulin-like growth factor-binding protein 5	U = 0.000	0.029	0.174	0.082190587
IGF-BP-6	Insulin-like growth factor-binding protein 6	t = 2.375	0.0552	0.278905263	0.356706572
IGF-I	Insulin-like growth factor 1	t = 0.385	0.713	0.777818182	0.871173382
IGF-II	**Insulin-like growth factor 2**	t = -0.701	0.51	0.589879518	1.79346011
IL10	Interleukin-10	U = 4.000	0.343	0.539803279	2.17023141
IL12-p40/p70	**Interleukin-12 subunit beta**	U = 4.000	0.343	0.531096774	0.748800682
IL12-p70	Interleukin-12 subunit alpha	t = 1.628	0.155	0.450909091	0.545764089
IL13	Interleukin-13	t = 1.689	0.142	0.439741935	0.476935722
IL-15	Interleukin-15	t = -1.648	0.15	0.45	399.0667508
IL-17	Interleukin-17A	U = 4.000	0.343	0.522666667	2.826352885
IL-17B R	**Interleukin-17 receptor B**	t = -1.582	0.165	0.44	12.93322457

IL1-alpha	Interleukin-1 alpha	t = 2.237	0.0667	0.2668	0.407948186
IL1-beta	Interleukin-1 beta	U = 4.000	0.343	0.5145	1.641501978
IL-2	Interleukin-2	t = 3.122	0.0205	0.1968	0.268452492
IL-3	Interleukin-3	U = 4.000	0.343	0.506584615	1.408417814
IL-3 Rb	**Cytokine receptor common subunit beta**	t = 3.319	0.016	0.170666667	0.259341267
IL-4	Interleukin-4	U = 0.000	0.029	0.163764706	0.144573775
IL-5	Interleukin-5	U = 5.500	0.486	0.576	1.387367715
IL-6	Interleukin-6	U = 7.000	0.886	0.945066667	2.768347848
IL-7	Interleukin-7	t = -1.191	0.278	0.494222222	3.343629353
IL-9	**Interleukin-9**	t = 3.084	0.0216	0.188509091	0.27354691
I-TAC	C-X-C motif chemokine 11	t = -0.989	0.361	0.50226087	2.54948158
KC	**Growth-regulated alpha protein**	t = 1.716	0.137	0.4384	0.454070233
Leptin	Leptin	t = 2.972	0.0249	0.183876923	0.00039845
Leptin R	**Leptin receptor**	t = 3.373	0.015	0.18	0.19175143
LIX	**C-X-C motif chemokine 5**	t = 0.712	0.503	0.588878049	0.770974487
L-Selectin	**L-selectin**	t = -0.0810	0.938	0.978782609	1.044704172
Lungkine	**C-X-C motif chemokine 15**	t = -1.858	0.113	0.43392	3.077990289
Lymphotactin	Lymphotactin	t = 0.903	0.402	0.536	0.652120889
MCP-1	C-C motif chemokine 2	t = 0.911	0.398	0.538140845	0.642811916
MCP-5	**C-C motif chemokine 12**	t = 1.038	0.339	0.570947368	0.597468689
M-CSF	Macrophage colony-stimulating factor 1	t = 1.586	0.164	0.449828571	0.532864653
MDC	C-C motif chemokine 22	t = -1.177	0.284	0.495709091	4.074747523
MIG	C-X-C motif chemokine 9	U= 0.000	0.029	0.154666667	0.00204976
MIP-1-alpha	C-C motif chemokine 3	t = 2.302	0.0609	0.265745455	0.378817372
MIP-1-gamma	**C-C motif chemokine 9**	t = 1.064	0.328	0.562285714	0.734915777
MIP-2	**C-X-C motif chemokine 2**	t = 0.797	0.456	0.58368	0.789007459
MIP-3-alpha	C-C motif chemokine 20	U = 4.000	0.343	0.498909091	2.121203724
MIP-3-beta	C-C motif chemokine 19	t = 0.983	0.363	0.497828571	0.606137853
MMP-2	**72 kDa type IV collagenase**	t = -1.304	0.24	0.48	3.541073851
MMP-3	**Stromelysin-1**	U = 8.000	1	1.010526316	6.936560232
Osteopontin	**Osteopontin**	t = -2.883	0.0279	0.191314286	3.833710361
Osteoporotegerin	**Tumor necrosis factor receptor superfamily member 11B**	U = 8.000	1	1	4.333477692
PF4	**Platelet factor 4**	t = 2.287	0.0622	0.259617391	0.563143877
Pro-MMP-9	**Matrix metalloproteinase-9**	t = -0.494	0.639	0.730285714	1.717687427
P-Selectin	**P-selectin**	t = 1.782	0.125	0.428571429	0.539184918
RANTES	C-C motif chemokine 5	U = 6.000	0.686	0.756965517	1.924556543
Resistin	**Resistin**	t = -0.843	0.432	0.560432432	1.592337008
SCF	Kit ligand	t = 12.460	0.0000163	0.0015648	0.069197739
SDF-1-alpha	**Stromal cell-derived factor 1**	t = 1.571	0.167	0.433297297	0.453353592
Shh-N	Sonic Hedgehog N-Terminal	t = -1.462	0.194	0.454243902	9.042914552

sTNF RI	Tumor necrosis factor receptor superfamily member 1A	U = 7.000	0.886	0.934681319	1.809589717
sTNF RII	Tumor necrosis factor receptor superfamily member 1B	t = 1.723	0.136	0.450206897	0.514783869
TARC	C-C motif chemokine 17	t = 0.855	0.425	0.55890411	0.691390998
TCA-3	**C-C motif chemokine 1**	t = 0.455	0.665	0.751058824	0.830863457
TECK	C-C motif chemokine 25	t = 0.790	0.46	0.581052632	0.646734739
Thymus CK-1	**Chemokine subfamily B Cys-X-Cys**	t = -1.231	0.265	0.489230769	1.871607785
TIMP-1	Metalloproteinase inhibitor 1	U = 4.000	0.343	0.491462687	0.914069023
TIMP-2	Metalloproteinase inhibitor 2	t = -1.368	0.22	0.469333333	3.645891367
TNF-alpha	Tumor necrosis factor	U = 4.000	0.343	0.484235294	1.986775267
TPO	Thrombopoietin	t = 0.772	0.469	0.584727273	0.680570634
TRANCE	**Tumor necrosis factor ligand superfamily member 11**	t = -1.315	0.236	0.482042553	2.782523147
TROY	**Tumor necrosis factor receptor superfamily member 19**	t = -1.254	0.257	0.483764706	3.781633437
TSLP	**Thymic stromal lymphopoietin**	t = -1.279	0.248	0.47616	4.382825633
VCAM-1	**Vascular cell adhesion protein 1**	U = 3.000	0.2	0.446511628	0.604978701
VEGF	Vascular endothelial growth factor A, long form	t = 5.927	0.00103	0.04944	0.02339249
VEGF R1	**Vascular endothelial growth factor receptor 1**	t = -1.216	0.27	0.489056604	2.664285468
VEGF R2	**Vascular endothelial growth factor receptor 2**	t = -1.489	0.187	0.4488	4.764540184
VEGF R3	**Vascular endothelial growth factor receptor 3**	t = -1.409	0.208	0.453818182	8.974223246
VEGF-D	Vascular endothelial growth factor D	t = -1.353	0.225	0.469565217	10.61462991

* Student's unpaired two-tailed t-test or Mann-Whitney U test were used to compare chemokine and cytokine expression in CTRL vs MDD group. Proteins were considered significantly different at $p < 0.05$. The q-values were calculated using the Benjamini–Hochberg false discovery rate (FDR) method. Fold Change (FC) were calculated as MDD/CTRL ratio.
